# The PRISMA Statement for Reporting Systematic Reviews and Meta-Analyses of Studies That Evaluate Health Care Interventions: Explanation and Elaboration

**DOI:** 10.1371/journal.pmed.1000100

**Published:** 2009-07-21

**Authors:** Alessandro Liberati, Douglas G. Altman, Jennifer Tetzlaff, Cynthia Mulrow, Peter C. Gøtzsche, John P. A. Ioannidis, Mike Clarke, P. J. Devereaux, Jos Kleijnen, David Moher

**Affiliations:** 1 Università di Modena e Reggio Emilia, Modena, Italy; 2 Centro Cochrane Italiano, Istituto Ricerche Farmacologiche Mario Negri, Milan, Italy; 3 Centre for Statistics in Medicine, University of Oxford, Oxford, United Kingdom; 4 Ottawa Methods Centre, Ottawa Hospital Research Institute, Ottawa, Ontario, Canada; 5 Annals of Internal Medicine, Philadelphia, Pennsylvania, United States of America; 6 The Nordic Cochrane Centre, Copenhagen, Denmark; 7 Department of Hygiene and Epidemiology, University of Ioannina School of Medicine, Ioannina, Greece; 8 UK Cochrane Centre, Oxford, United Kingdom; 9 School of Nursing and Midwifery, Trinity College, Dublin, Ireland; 10 Departments of Medicine, Clinical Epidemiology and Biostatistics, McMaster University, Hamilton, Ontario, Canada; 11 Kleijnen Systematic Reviews Ltd, York, United Kingdom; 12 School for Public Health and Primary Care (CAPHRI), University of Maastricht, Maastricht, The Netherlands; 13 Department of Epidemiology and Community Medicine, Faculty of Medicine, Ottawa, Ontario, Canada

## Abstract

Systematic reviews and meta-analyses are essential to summarize evidence relating to efficacy and safety of health care interventions accurately and reliably. The clarity and transparency of these reports, however, is not optimal. Poor reporting of systematic reviews diminishes their value to clinicians, policy makers, and other users.

Since the development of the QUOROM (*QU*ality *O*f *R*eporting *O*f *M*eta-analysis) Statement—a reporting guideline published in 1999—there have been several conceptual, methodological, and practical advances regarding the conduct and reporting of systematic reviews and meta-analyses. Also, reviews of published systematic reviews have found that key information about these studies is often poorly reported. Realizing these issues, an international group that included experienced authors and methodologists developed PRISMA (Preferred Reporting Items for Systematic reviews and Meta-Analyses) as an evolution of the original QUOROM guideline for systematic reviews and meta-analyses of evaluations of health care interventions.

The PRISMA Statement consists of a 27-item checklist and a four-phase flow diagram. The checklist includes items deemed essential for transparent reporting of a systematic review. In this Explanation and Elaboration document, we explain the meaning and rationale for each checklist item. For each item, we include an example of good reporting and, where possible, references to relevant empirical studies and methodological literature. The PRISMA Statement, this document, and the associated Web site (http://www.prisma-statement.org/) should be helpful resources to improve reporting of systematic reviews and meta-analyses.

## Abstract 2

Alessandro Liberati and colleagues present an Explanation and Elaboration of the PRISMA Statement, updated guidelines for the reporting of systematic reviews and meta-analyses.

## Introduction

Systematic reviews and meta-analyses are essential tools for summarizing evidence accurately and reliably. They help clinicians keep up-to-date; provide evidence for policy makers to judge risks, benefits, and harms of health care behaviors and interventions; gather together and summarize related research for patients and their carers; provide a starting point for clinical practice guideline developers; provide summaries of previous research for funders wishing to support new research [1]; and help editors judge the merits of publishing reports of new studies [2]. Recent data suggest that at least 2,500 new systematic reviews reported in English are indexed in MEDLINE annually [3].

Unfortunately, there is considerable evidence that key information is often poorly reported in systematic reviews, thus diminishing their potential usefulness [3],[4],[5],[6]. As is true for all research, systematic reviews should be reported fully and transparently to allow readers to assess the strengths and weaknesses of the investigation [7]. That rationale led to the development of the QUOROM (*QU*ality *O*f *R*eporting *O*f *M*eta-analyses) Statement; those detailed reporting recommendations were published in 1999 [8]. In this paper we describe the updating of that guidance. Our aim is to ensure clear presentation of what was planned, done, and found in a systematic review.

Terminology used to describe systematic reviews and meta-analyses has evolved over time and varies across different groups of researchers and authors (see Box 1). In this document we adopt the definitions used by the Cochrane Collaboration [9]. A systematic review attempts to collate all empirical evidence that fits pre-specified eligibility criteria to answer a specific research question. It uses explicit, systematic methods that are selected to minimize bias, thus providing reliable findings from which conclusions can be drawn and decisions made. Meta-analysis is the use of statistical methods to summarize and combine the results of independent studies. Many systematic reviews contain meta-analyses, but not all.

**Box 1. Terminology**

The terminology used to describe systematic reviews and meta-analyses has evolved over time and varies between fields. Different terms have been used by different groups, such as educators and psychologists. The conduct of a systematic review comprises several explicit and reproducible steps, such as identifying all likely relevant records, selecting eligible studies, assessing the risk of bias, extracting data, qualitative synthesis of the included studies, and possibly meta-analyses.

Initially this entire process was termed a meta-analysis and was so defined in the QUOROM Statement [8]. More recently, especially in health care research, there has been a trend towards preferring the term systematic review. If quantitative synthesis is performed, this last stage alone is referred to as a meta-analysis. The Cochrane Collaboration uses this terminology [9], under which a meta-analysis, if performed, is a component of a systematic review. Regardless of the question addressed and the complexities involved, it is always possible to complete a systematic review of existing data, but not always possible, or desirable, to quantitatively synthesize results, due to clinical, methodological, or statistical differences across the included studies. Conversely, with prospective accumulation of studies and datasets where the plan is eventually to combine them, the term “(prospective) meta-analysis” may make more sense than “systematic review.”

For retrospective efforts, one possibility is to use the term systematic review for the whole process up to the point when one decides whether to perform a quantitative synthesis. If a quantitative synthesis is performed, some researchers refer to this as a meta-analysis. This definition is similar to that found in the current edition of the *Dictionary of Epidemiology* [183].

While we recognize that the use of these terms is inconsistent and there is residual disagreement among the members of the panel working on PRISMA, we have adopted the definitions used by the Cochrane Collaboration [9].

**Systematic review:** A systematic review attempts to collate all empirical evidence that fits pre-specified eligibility criteria to answer a specific research question. It uses explicit, systematic methods that are selected with a view to minimizing bias, thus providing reliable findings from which conclusions can be drawn and decisions made [184],[185]. The key characteristics of a systematic review are: (a) a clearly stated set of objectives with an explicit, reproducible methodology; (b) a systematic search that attempts to identify all studies that would meet the eligibility criteria; (c) an assessment of the validity of the findings of the included studies, for example through the assessment of risk of bias; and (d) systematic presentation, and synthesis, of the characteristics and findings of the included studies.

**Meta-analysis:** Meta-analysis is the use of statistical techniques to integrate and summarize the results of included studies. Many systematic reviews contain meta-analyses, but not all. By combining information from all relevant studies, meta-analyses can provide more precise estimates of the effects of health care than those derived from the individual studies included within a review.

## The QUOROM Statement and Its Evolution into PRISMA

The QUOROM Statement, developed in 1996 and published in 1999 [8], was conceived as a reporting guidance for authors reporting a meta-analysis of randomized trials. Since then, much has happened. First, knowledge about the conduct and reporting of systematic reviews has expanded considerably. For example, The Cochrane Library's Methodology Register (which includes reports of studies relevant to the methods for systematic reviews) now contains more than 11,000 entries (March 2009). Second, there have been many conceptual advances, such as “outcome-level” assessments of the risk of bias [10],[11], that apply to systematic reviews. Third, authors have increasingly used systematic reviews to summarize evidence other than that provided by randomized trials.

However, despite advances, the quality of the conduct and reporting of systematic reviews remains well short of ideal [3],[4],[5],[6]. All of these issues prompted the need for an update and expansion of the QUOROM Statement. Of note, recognizing that the updated statement now addresses the above conceptual and methodological issues and may also have broader applicability than the original QUOROM Statement, we changed the name of the reporting guidance to PRISMA (Preferred Reporting Items for Systematic reviews and Meta-Analyses).

## Development of PRISMA

**Figure 1:**
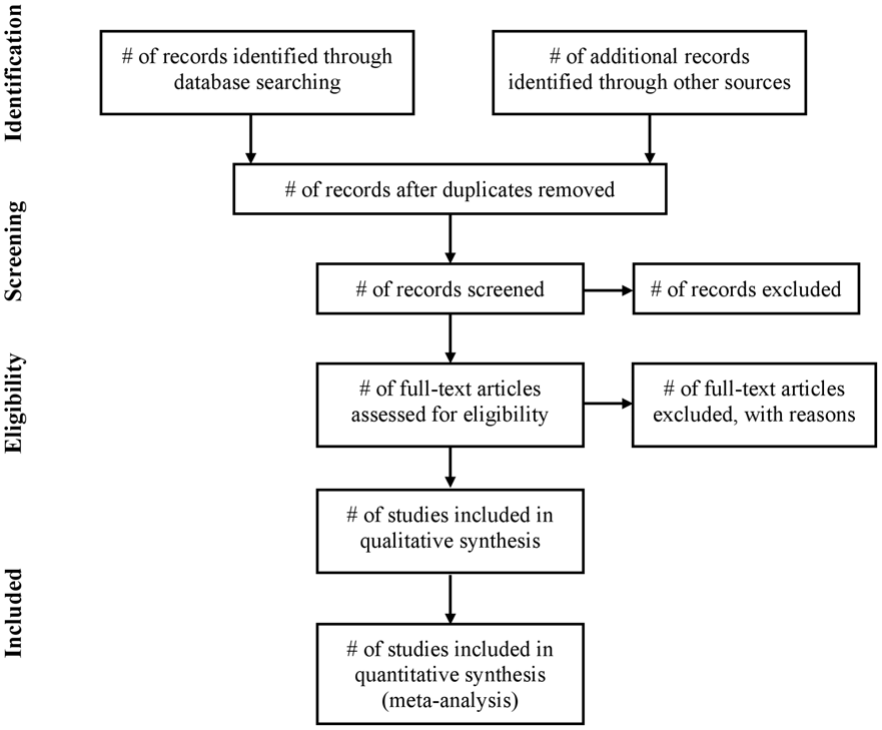
Flow of information through the different phases of a systematic review.

The PRISMA Statement was developed by a group of 29 review authors, methodologists, clinicians, medical editors, and consumers [12]. They attended a three-day meeting in 2005 and participated in extensive post-meeting electronic correspondence. A consensus process that was informed by evidence, whenever possible, was used to develop a 27-item checklist (Table 1; see also Text S1 for a downloadable template checklist for researchers to re-use) and a four-phase flow diagram (Figure 1; see Figure S1 for a downloadable template document for researchers to re-use). Items deemed essential for transparent reporting of a systematic review were included in the checklist. The flow diagram originally proposed by QUOROM was also modified to show numbers of identified records, excluded articles, and included studies. After 11 revisions the group approved the checklist, flow diagram, and this explanatory paper.

The PRISMA Statement itself provides further details regarding its background and development [12]. This accompanying Explanation and Elaboration document explains the meaning and rationale for each checklist item. A few PRISMA Group participants volunteered to help draft specific items for this document, and four of these (DGA, AL, DM, and JT) met on several occasions to further refine the document, which was circulated and ultimately approved by the larger PRISMA Group.

**Table 1:** Checklist of items to include when reporting a systematic review (with or without meta-analysis).

Section/Topic	#	Checklist Item	Reported on Page #
**TITLE**			
Title	1	Identify the report as a systematic review, meta-analysis, or both.	
**ABSTRACT**			
Structured summary	2	Provide a structured summary including, as applicable: background; objectives; data sources; study eligibility criteria, participants, and interventions; study appraisal and synthesis methods; results; limitations; conclusions and implications of key findings; systematic review registration number.	
**INTRODUCTION**			
Rationale	3	Describe the rationale for the review in the context of what is already known.	
Objectives	4	Provide an explicit statement of questions being addressed with reference to participants, interventions, comparisons, outcomes, and study design (PICOS).	
**METHODS**			
Protocol and registration	5	Indicate if a review protocol exists, if and where it can be accessed (e.g., Web address), and, if available, provide registration information including registration number.	
Eligibility criteria	6	Specify study characteristics (e.g., PICOS, length of follow-up) and report characteristics (e.g., years considered, language, publication status) used as criteria for eligibility, giving rationale.	
Information sources	7	Describe all information sources (e.g., databases with dates of coverage, contact with study authors to identify additional studies) in the search and date last searched.	
Search	8	Present full electronic search strategy for at least one database, including any limits used, such that it could be repeated.	
Study selection	9	State the process for selecting studies (i.e., screening, eligibility, included in systematic review, and, if applicable, included in the meta-analysis).	
Data collection process	10	Describe method of data extraction from reports (e.g., piloted forms, independently, in duplicate) and any processes for obtaining and confirming data from investigators.	
Data items	11	List and define all variables for which data were sought (e.g., PICOS, funding sources) and any assumptions and simplifications made.	
Risk of bias in individual studies	12	Describe methods used for assessing risk of bias of individual studies (including specification of whether this was done at the study or outcome level), and how this information is to be used in any data synthesis.	
Summary measures	13	State the principal summary measures (e.g., risk ratio, difference in means).	
Synthesis of results	14	Describe the methods of handling data and combining results of studies, if done, including measures of consistency (e.g., I^2^) for each meta-analysis.	
Risk of bias across studies	15	Specify any assessment of risk of bias that may affect the cumulative evidence (e.g., publication bias, selective reporting within studies).	
Additional analyses	16	Describe methods of additional analyses (e.g., sensitivity or subgroup analyses, meta-regression), if done, indicating which were pre-specified.	
**RESULTS**			
Study selection	17	Give numbers of studies screened, assessed for eligibility, and included in the review, with reasons for exclusions at each stage, ideally with a flow diagram.	
Study characteristics	18	For each study, present characteristics for which data were extracted (e.g., study size, PICOS, follow-up period) and provide the citations.	
Risk of bias within studies	19	Present data on risk of bias of each study and, if available, any outcome-level assessment (see Item 12).	
Results of individual studies	20	For all outcomes considered (benefits or harms), present, for each study: (a) simple summary data for each intervention group and (b) effect estimates and confidence intervals, ideally with a forest plot.	
Synthesis of results	21	Present results of each meta-analysis done, including confidence intervals and measures of consistency.	
Risk of bias across studies	22	Present results of any assessment of risk of bias across studies (see Item 15).	
Additional analysis	23	Give results of additional analyses, if done (e.g., sensitivity or subgroup analyses, meta-regression [see Item 16]).	
**DISCUSSION**			
Summary of evidence	24	Summarize the main findings including the strength of evidence for each main outcome; consider their relevance to key groups (e.g., health care providers, users, and policy makers).	
Limitations	25	Discuss limitations at study and outcome level (e.g., risk of bias), and at review level (e.g., incomplete retrieval of identified research, reporting bias).	
Conclusions	26	Provide a general interpretation of the results in the context of other evidence, and implications for future research.	
**FUNDING**			
Funding	27	Describe sources of funding for the systematic review and other support (e.g., supply of data); role of funders for the systematic review.	

## Scope of PRISMA

PRISMA focuses on ways in which authors can ensure the transparent and complete reporting of systematic reviews and meta-analyses. It does not address directly or in a detailed manner the conduct of systematic reviews, for which other guides are available [13],[14],[15],[16].

We developed the PRISMA Statement and this explanatory document to help authors report a wide array of systematic reviews to assess the benefits and harms of a health care intervention. We consider most of the checklist items relevant when reporting systematic reviews of non-randomized studies assessing the benefits and harms of interventions. However, we recognize that authors who address questions relating to etiology, diagnosis, or prognosis, for example, and who review epidemiological or diagnostic accuracy studies may need to modify or incorporate additional items for their systematic reviews.

## How To Use This Paper

We modeled this Explanation and Elaboration document after those prepared for other reporting guidelines [17],[18],[19]. To maximize the benefit of this document, we encourage people to read it in conjunction with the PRISMA Statement [11].

We present each checklist item and follow it with a published exemplar of good reporting for that item. (We edited some examples by removing citations or Web addresses, or by spelling out abbreviations.) We then explain the pertinent issue, the rationale for including the item, and relevant evidence from the literature, whenever possible. No systematic search was carried out to identify exemplars and evidence. We also include seven Boxes that provide a more comprehensive explanation of certain thematic aspects of the methodology and conduct of systematic reviews.

Although we focus on a minimal list of items to consider when reporting a systematic review, we indicate places where additional information is desirable to improve transparency of the review process. We present the items numerically from 1 to 27; however, authors need not address items in this particular order in their reports. Rather, what is important is that the information for each item is given somewhere within the report.

## The PRISMA Checklist

### TITLE and ABSTRACT

#### Item 1: TITLE

Identify the report as a systematic review, meta-analysis, or both.

**Examples.** “Recurrence rates of video-assisted thoracoscopic versus open surgery in the prevention of recurrent pneumothoraces: a systematic review of randomised and non-randomised trials” [20]

“Mortality in randomized trials of antioxidant supplements for primary and secondary prevention: systematic review and meta-analysis” [21]

#### Explanation

Authors should identify their report as a systematic review or meta-analysis. Terms such as “review” or “overview” do not describe for readers whether the review was systematic or whether a meta-analysis was performed. A recent survey found that 50% of 300 authors did not mention the terms “systematic review” or “meta-analysis” in the title or abstract of their systematic review [3]. Although sensitive search strategies have been developed to identify systematic reviews [22], inclusion of the terms systematic review or meta-analysis in the title may improve indexing and identification.

We advise authors to use informative titles that make key information easily accessible to readers. Ideally, a title reflecting the PICOS approach (participants, interventions, comparators, outcomes, and study design) (see Item 11 and Box 2) may help readers as it provides key information about the scope of the review. Specifying the design(s) of the studies included, as shown in the examples, may also help some readers and those searching databases.

**Box 2. Helping To Develop the Research Question(s): The PICOS Approach**

Formulating relevant and precise questions that can be answered in a systematic review can be complex and time consuming. A structured approach for framing questions that uses five components may help facilitate the process. This approach is commonly known by the acronym “PICOS” where each letter refers to a component: the patient population or the disease being addressed (P), the interventions or exposure (I), the comparator group (C), the outcome or endpoint (O), and the study design chosen (S) [186]. Issues relating to PICOS impact several PRISMA items (i.e., Items 6, 8, 9, 10, 11, and 18).

Providing information about the **population** requires a precise definition of a group of participants (often patients), such as men over the age of 65 years, their defining characteristics of interest (often disease), and possibly the setting of care considered, such as an acute care hospital.

The **interventions** (exposures) under consideration in the systematic review need to be transparently reported. For example, if the reviewers answer a question regarding the association between a woman's prenatal exposure to folic acid and subsequent offspring's neural tube defects, reporting the dose, frequency, and duration of folic acid used in different studies is likely to be important for readers to interpret the review's results and conclusions. Other interventions (exposures) might include diagnostic, preventative, or therapeutic treatments, arrangements of specific processes of care, lifestyle changes, psychosocial or educational interventions, or risk factors.

Clearly reporting the **comparator** (control) group intervention(s), such as usual care, drug, or placebo, is essential for readers to fully understand the selection criteria of primary studies included in systematic reviews, and might be a source of heterogeneity investigators have to deal with. Comparators are often very poorly described. Clearly reporting what the intervention is compared with is very important and may sometimes have implications for the inclusion of studies in a review—many reviews compare with “standard care,” which is otherwise undefined; this should be properly addressed by authors.

The **outcomes** of the intervention being assessed, such as mortality, morbidity, symptoms, or quality of life improvements, should be clearly specified as they are required to interpret the validity and generalizability of the systematic review's results.

Finally, the type of **study design(s)** included in the review should be reported. Some reviews only include reports of randomized trials whereas others have broader design criteria and include randomized trials and certain types of observational studies. Still other reviews, such as those specifically answering questions related to harms, may include a wide variety of designs ranging from cohort studies to case reports. Whatever study designs are included in the review, these should be reported.

Independently from how difficult it is to identify the components of the research question, the important point is that a structured approach is preferable, and this extends beyond systematic reviews of effectiveness. Ideally the PICOS criteria should be formulated a priori, in the systematic review's protocol, although some revisions might be required due to the iterative nature of the review process. Authors are encouraged to report their PICOS criteria and whether any modifications were made during the review process. A useful example in this realm is the Appendix of the “Systematic Reviews of Water Fluoridation” undertaken by the Centre for Reviews and Dissemination [187].

Some journals recommend “indicative titles” that indicate the topic matter of the review, while others require declarative titles that give the review's main conclusion. Busy practitioners may prefer to see the conclusion of the review in the title, but declarative titles can oversimplify or exaggerate findings. Thus, many journals and methodologists prefer indicative titles as used in the examples above.

#### Item 2: STRUCTURED SUMMARY

Provide a structured summary including, as applicable: background; objectives; data sources; study eligibility criteria, participants, and interventions; study appraisal and synthesis methods; results; limitations; conclusions and implications of key findings; funding for the systematic review; and systematic review registration number.

**Example.** “*Context*: The role and dose of oral vitamin D supplementation in nonvertebral fracture prevention have not been well established.

*Objective*: To estimate the effectiveness of vitamin D supplementation in preventing hip and nonvertebral fractures in older persons.

*Data Sources*: A systematic review of English and non-English articles using MEDLINE and the Cochrane Controlled Trials Register (1960–2005), and EMBASE (1991–2005). Additional studies were identified by contacting clinical experts and searching bibliographies and abstracts presented at the American Society for Bone and Mineral Research (1995–2004). Search terms included randomized controlled trial (RCT), controlled clinical trial, random allocation, double-blind method, cholecalciferol, ergocalciferol, 25-hydroxyvitamin D, fractures, humans, elderly, falls, and bone density.

*Study Selection*: Only double-blind RCTs of oral vitamin D supplementation (cholecalciferol, ergocalciferol) with or without calcium supplementation vs calcium supplementation or placebo in older persons (>60 years) that examined hip or nonvertebral fractures were included.

*Data Extraction*: Independent extraction of articles by 2 authors using predefined data fields, including study quality indicators.

*Data Synthesis*: All pooled analyses were based on random-effects models. Five RCTs for hip fracture (n = 9294) and 7 RCTs for nonvertebral fracture risk (n = 9820) met our inclusion criteria. All trials used cholecalciferol. Heterogeneity among studies for both hip and nonvertebral fracture prevention was observed, which disappeared after pooling RCTs with low-dose (400 IU/d) and higher-dose vitamin D (700–800 IU/d), separately. A vitamin D dose of 700 to 800 IU/d reduced the relative risk (RR) of hip fracture by 26% (3 RCTs with 5572 persons; pooled RR, 0.74; 95% confidence interval [CI], 0.61–0.88) and any nonvertebral fracture by 23% (5 RCTs with 6098 persons; pooled RR, 0.77; 95% CI, 0.68–0.87) vs calcium or placebo. No significant benefit was observed for RCTs with 400 IU/d vitamin D (2 RCTs with 3722 persons; pooled RR for hip fracture, 1.15; 95% CI, 0.88–1.50; and pooled RR for any nonvertebral fracture, 1.03; 95% CI, 0.86–1.24).

*Conclusions*: Oral vitamin D supplementation between 700 to 800 IU/d appears to reduce the risk of hip and any nonvertebral fractures in ambulatory or institutionalized elderly persons. An oral vitamin D dose of 400 IU/d is not sufficient for fracture prevention.” [23]

#### Explanation

Abstracts provide key information that enables readers to understand the scope, processes, and findings of a review and to decide whether to read the full report. The abstract may be all that is readily available to a reader, for example, in a bibliographic database. The abstract should present a balanced and realistic assessment of the review's findings that mirrors, albeit briefly, the main text of the report.

We agree with others that the quality of reporting in abstracts presented at conferences and in journal publications needs improvement [24],[25]. While we do not uniformly favor a specific format over another, we generally recommend structured abstracts. Structured abstracts provide readers with a series of headings pertaining to the purpose, conduct, findings, and conclusions of the systematic review being reported [26],[27]. They give readers more complete information and facilitate finding information more easily than unstructured abstracts [28],[29],[30],[31],[32].

A highly structured abstract of a systematic review could include the following headings: Context (or Background); Objective (or Purpose); Data Sources; Study Selection (or Eligibility Criteria); Study Appraisal and Synthesis Methods (or Data Extraction and Data Synthesis); Results; Limitations; and Conclusions (or Implications). Alternatively, a simpler structure could cover but collapse some of the above headings (e.g., label Study Selection and Study Appraisal as Review Methods) or omit some headings such as Background and Limitations.

In the highly structured abstract mentioned above, authors use the *Background* heading to set the context for readers and explain the importance of the review question. Under the *Objectives* heading, they ideally use elements of PICOS (see Box 2) to state the primary objective of the review. Under a *Data Sources* heading, they summarize sources that were searched, any language or publication type restrictions, and the start and end dates of searches. *Study Selection* statements then ideally describe who selected studies using what inclusion criteria. *Data Extraction Methods* statements describe appraisal methods during data abstraction and the methods used to integrate or summarize the data. The *Data Synthesis* section is where the main results of the review are reported. If the review includes meta-analyses, authors should provide numerical results with confidence intervals for the most important outcomes. Ideally, they should specify the amount of evidence in these analyses (numbers of studies and numbers of participants). Under a *Limitations* heading, authors might describe the most important weaknesses of included studies as well as limitations of the review process. Then authors should provide clear and balanced *Conclusions* that are closely linked to the objective and findings of the review. Additionally, it would be helpful if authors included some information about funding for the review. Finally, although protocol registration for systematic reviews is still not common practice, if authors have registered their review or received a registration number, we recommend providing the registration information at the end of the abstract.

Taking all the above considerations into account, the intrinsic tension between the goal of completeness of the abstract and its keeping into the space limit often set by journal editors is recognized as a major challenge.

### INTRODUCTION

#### Item 3: RATIONALE

Describe the rationale for the review in the context of what is already known.

**Example.** “Reversing the trend of increasing weight for height in children has proven difficult. It is widely accepted that increasing energy expenditure and reducing energy intake form the theoretical basis for management. Therefore, interventions aiming to increase physical activity and improve diet are the foundation of efforts to prevent and treat childhood obesity. Such lifestyle interventions have been supported by recent systematic reviews, as well as by the Canadian Paediatric Society, the Royal College of Paediatrics and Child Health, and the American Academy of Pediatrics. However, these interventions are fraught with poor adherence. Thus, school-based interventions are theoretically appealing because adherence with interventions can be improved. Consequently, many local governments have enacted or are considering policies that mandate increased physical activity in schools, although the effect of such interventions on body composition has not been assessed.” [33]

#### Explanation

Readers need to understand the rationale behind the study and what the systematic review may add to what is already known. Authors should tell readers whether their report is a new systematic review or an update of an existing one. If the review is an update, authors should state reasons for the update, including what has been added to the evidence base since the previous version of the review.

An ideal background or introduction that sets context for readers might include the following. First, authors might define the importance of the review question from different perspectives (e.g., public health, individual patient, or health policy). Second, authors might briefly mention the current state of knowledge and its limitations. As in the above example, information about the effects of several different interventions may be available that helps readers understand why potential relative benefits or harms of particular interventions need review. Third, authors might whet readers' appetites by clearly stating what the review aims to add. They also could discuss the extent to which the limitations of the existing evidence base may be overcome by the review.

#### Item 4: OBJECTIVES

Provide an explicit statement of questions being addressed with reference to participants, interventions, comparisons, outcomes, and study design (PICOS).

**Example.** “To examine whether topical or intraluminal antibiotics reduce catheter-related bloodstream infection, we reviewed randomized, controlled trials that assessed the efficacy of these antibiotics for primary prophylaxis against catheter-related bloodstream infection and mortality compared with no antibiotic therapy in adults undergoing hemodialysis.” [34]

#### Explanation

The questions being addressed, and the rationale for them, are one of the most critical parts of a systematic review. They should be stated precisely and explicitly so that readers can understand quickly the review's scope and the potential applicability of the review to their interests [35]. Framing questions so that they include the following five “PICOS” components may improve the explicitness of review questions: (1) the patient population or disease being addressed (P), (2) the interventions or exposure of interest (I), (3) the comparators (C), (4) the main outcome or endpoint of interest (O), and (5) the study designs chosen (S). For more detail regarding PICOS, see Box 2.

Good review questions may be narrowly focused or broad, depending on the overall objectives of the review. Sometimes broad questions might increase the applicability of the results and facilitate detection of bias, exploratory analyses, and sensitivity analyses [35],[36]. Whether narrowly focused or broad, precisely stated review objectives are critical as they help define other components of the review process such as the eligibility criteria (Item 6) and the search for relevant literature (Items 7 and 8).

### METHODS

#### Item 5: PROTOCOL AND REGISTRATION

Indicate if a review protocol exists, if and where it can be accessed (e.g., Web address) and, if available, provide registration information including the registration number.

**Example.** “Methods of the analysis and inclusion criteria were specified in advance and documented in a protocol.” [37]

#### Explanation

A protocol is important because it pre-specifies the objectives and methods of the systematic review. For instance, a protocol specifies outcomes of primary interest, how reviewers will extract information about those outcomes, and methods that reviewers might use to quantitatively summarize the outcome data (see Item 13). Having a protocol can help restrict the likelihood of biased post hoc decisions in review methods, such as selective outcome reporting. Several sources provide guidance about elements to include in the protocol for a systematic review [16],[38],[39]. For meta-analyses of individual patient-level data, we advise authors to describe whether a protocol was explicitly designed and whether, when, and how participating collaborators endorsed it [40],[41].

Authors may modify protocols during the research, and readers should not automatically consider such modifications inappropriate. For example, legitimate modifications may extend the period of searches to include older or newer studies, broaden eligibility criteria that proved too narrow, or add analyses if the primary analyses suggest that additional ones are warranted. Authors should, however, describe the modifications and explain their rationale.

Although worthwhile protocol amendments are common, one must consider the effects that protocol modifications may have on the results of a systematic review, especially if the primary outcome is changed. Bias from selective outcome reporting in randomized trials has been well documented [42],[43]. An examination of 47 Cochrane reviews revealed indirect evidence for possible selective reporting bias for systematic reviews. Almost all (*n* = 43) contained a major change, such as the addition or deletion of outcomes, between the protocol and the full publication [44]. Whether (or to what extent) the changes reflected bias, however, was not clear. For example, it has been rather common not to describe outcomes that were not presented in any of the included studies.

Registration of a systematic review, typically with a protocol and registration number, is not yet common, but some opportunities exist [45],[46]. Registration may possibly reduce the risk of multiple reviews addressing the same question [45],[46],[47],[48], reduce publication bias, and provide greater transparency when updating systematic reviews. Of note, a survey of systematic reviews indexed in MEDLINE in November 2004 found that reports of protocol use had increased to about 46% [3] from 8% noted in previous surveys [49]. The improvement was due mostly to Cochrane reviews, which, by requirement, have a published protocol [3].

#### Item 6: ELIGIBILITY CRITERIA

Specify study characteristics (e.g., PICOS, length of follow-up) and report characteristics (e.g., years considered, language, publication status) used as criteria for eligibility, giving rationale.

**Examples.** *Types of studies*: “Randomised clinical trials studying the administration of hepatitis B vaccine to CRF [chronic renal failure] patients, with or without dialysis. No language, publication date, or publication status restrictions were imposed…”

*Types of participants*: “Participants of any age with CRF or receiving dialysis (haemodialysis or peritoneal dialysis) were considered. CRF was defined as serum creatinine greater than 200 µmol/L for a period of more than six months or individuals receiving dialysis (haemodialysis or peritoneal dialysis)…Renal transplant patients were excluded from this review as these individuals are immunosuppressed and are receiving immunosuppressant agents to prevent rejection of their transplanted organs, and they have essentially normal renal function…”

*Types of intervention*: “Trials comparing the beneficial and harmful effects of hepatitis B vaccines with adjuvant or cytokine co-interventions [and] trials comparing the beneficial and harmful effects of immunoglobulin prophylaxis. This review was limited to studies looking at active immunization. Hepatitis B vaccines (plasma or recombinant (yeast) derived) of all types, dose, and regimens versus placebo, control vaccine, or no vaccine…”

*Types of outcome measures*: “Primary outcome measures: Seroconversion, ie, proportion of patients with adequate anti-HBs response (>10 IU/L or Sample Ratio Units). Hepatitis B infections (as measured by hepatitis B core antigen (HBcAg) positivity or persistent HBsAg positivity), both acute and chronic. Acute (primary) HBV [hepatitis B virus] infections were defined as seroconversion to HBsAg positivity or development of IgM anti-HBc. Chronic HBV infections were defined as the persistence of HBsAg for more than six months or HBsAg positivity and liver biopsy compatible with a diagnosis or chronic hepatitis B. Secondary outcome measures: Adverse events of hepatitis B vaccinations…[and]…mortality.” [50]

#### Explanation

Knowledge of the eligibility criteria is essential in appraising the validity, applicability, and comprehensiveness of a review. Thus, authors should unambiguously specify eligibility criteria used in the review. Carefully defined eligibility criteria inform various steps of the review methodology. They influence the development of the search strategy and serve to ensure that studies are selected in a systematic and unbiased manner.

A study may be described in multiple reports, and one report may describe multiple studies. Therefore, we separate eligibility criteria into the following two components: study characteristics and report characteristics. Both need to be reported. Study eligibility criteria are likely to include the populations, interventions, comparators, outcomes, and study designs of interest (PICOS; see Box 2), as well as other study-specific elements, such as specifying a minimum length of follow-up. Authors should state whether studies will be excluded because they do not include (or report) specific outcomes to help readers ascertain whether the systematic review may be biased as a consequence of selective reporting [42],[43].

Report eligibility criteria are likely to include language of publication, publication status (e.g., inclusion of unpublished material and abstracts), and year of publication. Inclusion or not of non-English language literature [51],[52],[53],[54],[55], unpublished data, or older data can influence the effect estimates in meta-analyses [56],[57],[58],[59]. Caution may need to be exercised in including all identified studies due to potential differences in the risk of bias such as, for example, selective reporting in abstracts [60],[61],[62].

#### Item 7: INFORMATION SOURCES

Describe all information sources in the search (e.g., databases with dates of coverage, contact with study authors to identify additional studies) and date last searched.

**Example.** “Studies were identified by searching electronic databases, scanning reference lists of articles and consultation with experts in the field and drug companies…No limits were applied for language and foreign papers were translated. This search was applied to Medline (1966–Present), CancerLit (1975–Present), and adapted for Embase (1980–Present), Science Citation Index Expanded (1981–Present) and Pre-Medline electronic databases. Cochrane and DARE (Database of Abstracts of Reviews of Effectiveness) databases were reviewed…The last search was run on 19 June 2001. In addition, we handsearched contents pages of Journal of Clinical Oncology 2001, European Journal of Cancer 2001 and Bone 2001, together with abstracts printed in these journals 1999–2001. A limited update literature search was performed from 19 June 2001 to 31 December 2003.” [63]

#### Explanation

The National Library of Medicine's MEDLINE database is one of the most comprehensive sources of health care information in the world. Like any database, however, its coverage is not complete and varies according to the field. Retrieval from any single database, even by an experienced searcher, may be imperfect, which is why detailed reporting is important within the systematic review.

At a minimum, for each database searched, authors should report the database, platform, or provider (e.g., Ovid, Dialog, PubMed) and the start and end dates for the search of each database. This information lets readers assess the currency of the review, which is important because the publication time-lag outdates the results of some reviews [64]. This information should also make updating more efficient [65]. Authors should also report who developed and conducted the search [66].

In addition to searching databases, authors should report the use of supplementary approaches to identify studies, such as hand searching of journals, checking reference lists, searching trials registries or regulatory agency Web sites [67], contacting manufacturers, or contacting authors. Authors should also report if they attempted to acquire any missing information (e.g., on study methods or results) from investigators or sponsors; it is useful to describe briefly who was contacted and what unpublished information was obtained.

#### Item 8: SEARCH

Present the full electronic search strategy for at least one major database, including any limits used, such that it could be repeated.

**Examples.** *In text*: “We used the following search terms to search all trials registers and databases: immunoglobulin*; IVIG; sepsis; septic shock; septicaemia; and septicemia…” [68]

*In appendix*: “Search strategy: MEDLINE (OVID)

01. immunoglobulins/

02. immunoglobulin$.tw.

03. ivig.tw.

04. 1 or 2 or 3

05. sepsis/

06. sepsis.tw.

07. septic shock/

08. septic shock.tw.

09. septicemia/

10. septicaemia.tw.

11. septicemia.tw.

12. 5 or 6 or 7 or 8 or 9 or 10 or 11

13. 4 and 12

14. randomized controlled trials/

15. randomized-controlled-trial.pt.

16. controlled-clinical-trial.pt.

17. random allocation/

18. double-blind method/

19. single-blind method/

20. 14 or 15 or 16 or 17 or 18 or 19

21. exp clinical trials/

22. clinical-trial.pt.

23. (clin$ adj trial$).ti,ab.

24. ((singl$ or doubl$ or trebl$ or tripl$) adj (blind$)).ti,ab.

25. placebos/

26. placebo$.ti,ab.

27. random$.ti,ab.

28. 21 or 22 or 23 or 24 or 25 or 26 or 27

29. research design/

30. comparative study/

31. exp evaluation studies/

32. follow-up studies/

33. prospective studies/

34. (control$ or prospective$ or volunteer$).ti,ab.

35. 30 or 31 or 32 or 33 or 34

36. 20 or 28 or 29 or 35

37. 13 and 36” [68]

#### Explanation

The search strategy is an essential part of the report of any systematic review. Searches may be complicated and iterative, particularly when reviewers search unfamiliar databases or their review is addressing a broad or new topic. Perusing the search strategy allows interested readers to assess the comprehensiveness and completeness of the search, and to replicate it. Thus, we advise authors to report their full electronic search strategy for at least one major database. As an alternative to presenting search strategies for all databases, authors could indicate how the search took into account other databases searched, as index terms vary across databases. If different searches are used for different parts of a wider question (e.g., questions relating to benefits and questions relating to harms), we recommend authors provide at least one example of a strategy for each part of the objective [69]. We also encourage authors to state whether search strategies were peer reviewed as part of the systematic review process [70].

We realize that journal restrictions vary and that having the search strategy in the text of the report is not always feasible. We strongly encourage all journals, however, to find ways, such as a “Web extra,” appendix, or electronic link to an archive, to make search strategies accessible to readers. We also advise all authors to archive their searches so that (1) others may access and review them (e.g., replicate them or understand why their review of a similar topic did not identify the same reports), and (2) future updates of their review are facilitated.

Several sources provide guidance on developing search strategies [71],[72],[73]. Most searches have constraints, for example relating to limited time or financial resources, inaccessible or inadequately indexed reports and databases, unavailability of experts with particular language or database searching skills, or review questions for which pertinent evidence is not easy to find. Authors should be straightforward in describing their search constraints. Apart from the keywords used to identify or exclude records, they should report any additional limitations relevant to the search, such as language and date restrictions (see also eligibility criteria, Item 6) [51].

#### Item 9: STUDY SELECTION

State the process for selecting studies (i.e., for screening, for determining eligibility, for inclusion in the systematic review, and, if applicable, for inclusion in the meta-analysis).

**Example.** “Eligibility assessment…[was] performed independently in an unblinded standardized manner by 2 reviewers…Disagreements between reviewers were resolved by consensus.” [74]

#### Explanation

There is no standard process for selecting studies to include in a systematic review. Authors usually start with a large number of identified records from their search and sequentially exclude records according to eligibility criteria. We advise authors to report how they screened the retrieved records (typically a title and abstract), how often it was necessary to review the full text publication, and if any types of record (e.g., letters to the editor) were excluded. We also advise using the PRISMA flow diagram to summarize study selection processes (see Item 17; Box 3).

**Box 3. Identification of Study Reports and Data Extraction**

Comprehensive searches usually result in a large number of identified records, a much smaller number of studies included in the systematic review, and even fewer of these studies included in any meta-analyses. Reports of systematic reviews often provide little detail as to the methods used by the review team in this process. Readers are often left with what can be described as the “X-files” phenomenon, as it is unclear what occurs between the initial set of identified records and those finally included in the review.

Sometimes, review authors simply report the number of included studies; more often they report the initial number of identified records and the number of included studies. Rarely, although this is optimal for readers, do review authors report the number of identified records, the smaller number of potentially relevant studies, and the even smaller number of included studies, by outcome. Review authors also need to differentiate between the number of reports and studies. Often there will not be a 1∶1 ratio of reports to studies and this information needs to be described in the systematic review report.

Ideally, the identification of study reports should be reported as text in combination with use of the PRISMA flow diagram. While we recommend use of the flow diagram, a small number of reviews might be particularly simple and can be sufficiently described with a few brief sentences of text. More generally, review authors will need to report the process used for each step: screening the identified records; examining the full text of potentially relevant studies (and reporting the number that could not be obtained); and applying eligibility criteria to select the included studies.

Such descriptions should also detail how potentially eligible records were promoted to the next stage of the review (e.g., full text screening) and to the final stage of this process, the included studies. Often review teams have three response options for excluding records or promoting them to the next stage of the winnowing process: “yes,” “no,” and “maybe.”

Similarly, some detail should be reported on who participated and how such processes were completed. For example, a single person may screen the identified records while a second person independently examines a small sample of them. The entire winnowing process is one of “good book keeping” whereby interested readers should be able to work backwards from the included studies to come up with the same numbers of identified records.

There is often a paucity of information describing the data extraction processes in reports of systematic reviews. Authors may simply report that “relevant” data were extracted from each included study with little information about the processes used for data extraction. It may be useful for readers to know whether a systematic review's authors developed, a priori or not, a data extraction form, whether multiple forms were used, the number of questions, whether the form was pilot tested, and who completed the extraction. For example, it is important for readers to know whether one or more people extracted data, and if so, whether this was completed independently, whether “consensus” data were used in the analyses, and if the review team completed an informal training exercise or a more formal reliability exercise.

Efforts to enhance objectivity and avoid mistakes in study selection are important. Thus authors should report whether each stage was carried out by one or several people, who these people were, and, whenever multiple independent investigators performed the selection, what the process was for resolving disagreements. The use of at least two investigators may reduce the possibility of rejecting relevant reports [75]. The benefit may be greatest for topics where selection or rejection of an article requires difficult judgments [76]. For these topics, authors should ideally tell readers the level of inter-rater agreement, how commonly arbitration about selection was required, and what efforts were made to resolve disagreements (e.g., by contact with the authors of the original studies).

#### Item 10: DATA COLLECTION PROCESS

Describe the method of data extraction from reports (e.g., piloted forms, independently by two reviewers) and any processes for obtaining and confirming data from investigators.

**Example.** “We developed a data extraction sheet (based on the Cochrane Consumers and Communication Review Group's data extraction template), pilot-tested it on ten randomly-selected included studies, and refined it accordingly. One review author extracted the following data from included studies and the second author checked the extracted data…Disagreements were resolved by discussion between the two review authors; if no agreement could be reached, it was planned a third author would decide. We contacted five authors for further information. All responded and one provided numerical data that had only been presented graphically in the published paper.” [77]

#### Explanation

Reviewers extract information from each included study so that they can critique, present, and summarize evidence in a systematic review. They might also contact authors of included studies for information that has not been, or is unclearly, reported. In meta-analysis of individual patient data, this phase involves collection and scrutiny of detailed raw databases. The authors should describe these methods, including any steps taken to reduce bias and mistakes during data collection and data extraction [78] (Box 3).

Some systematic reviewers use a data extraction form that could be reported as an appendix or “Web extra” to their report. These forms could show the reader what information reviewers sought (see Item 11) and how they extracted it. Authors could tell readers if the form was piloted. Regardless, we advise authors to tell readers who extracted what data, whether any extractions were completed in duplicate, and, if so, whether duplicate abstraction was done independently and how disagreements were resolved.

Published reports of the included studies may not provide all the information required for the review. Reviewers should describe any actions they took to seek additional information from the original researchers (see Item 7). The description might include how they attempted to contact researchers, what they asked for, and their success in obtaining the necessary information. Authors should also tell readers when individual patient data were sought from the original researchers [41] (see Item 11) and indicate the studies for which such data were used in the analyses. The reviewers ideally should also state whether they confirmed the accuracy of the information included in their review with the original researchers, for example, by sending them a copy of the draft review [79].

Some studies are published more than once. Duplicate publications may be difficult to ascertain, and their inclusion may introduce bias [80],[81]. We advise authors to describe any steps they used to avoid double counting and piece together data from multiple reports of the same study (e.g., juxtaposing author names, treatment comparisons, sample sizes, or outcomes). We also advise authors to indicate whether all reports on a study were considered, as inconsistencies may reveal important limitations. For example, a review of multiple publications of drug trials showed that reported study characteristics may differ from report to report, including the description of the design, number of patients analyzed, chosen significance level, and outcomes [82]. Authors ideally should present any algorithm that they used to select data from overlapping reports and any efforts they used to solve logical inconsistencies across reports.

#### Item 11: DATA ITEMS

List and define all variables for which data were sought (e.g., PICOS, funding sources), and any assumptions and simplifications made.

**Examples.** “Information was extracted from each included trial on: (1) characteristics of trial participants (including age, stage and severity of disease, and method of diagnosis), and the trial's inclusion and exclusion criteria; (2) type of intervention (including type, dose, duration and frequency of the NSAID [non-steroidal anti-inflammatory drug]; versus placebo or versus the type, dose, duration and frequency of another NSAID; or versus another pain management drug; or versus no treatment); (3) type of outcome measure (including the level of pain reduction, improvement in quality of life score (using a validated scale), effect on daily activities, absence from work or school, length of follow up, unintended effects of treatment, number of women requiring more invasive treatment).” [83]

#### Explanation

It is important for readers to know what information review authors sought, even if some of this information was not available [84]. If the review is limited to reporting only those variables that were obtained, rather than those that were deemed important but could not be obtained, bias might be introduced and the reader might be misled. It is therefore helpful if authors can refer readers to the protocol (see Item 5), and archive their extraction forms (see Item 10), including definitions of variables. The published systematic review should include a description of the processes used with, if relevant, specification of how readers can get access to additional materials.

We encourage authors to report whether some variables were added after the review started. Such variables might include those found in the studies that the reviewers identified (e.g., important outcome measures that the reviewers initially overlooked). Authors should describe the reasons for adding any variables to those already pre-specified in the protocol so that readers can understand the review process.

We advise authors to report any assumptions they made about missing or unclear information and to explain those processes. For example, in studies of women aged 50 or older it is reasonable to assume that none were pregnant, even if this is not reported. Likewise, review authors might make assumptions about the route of administration of drugs assessed. However, special care should be taken in making assumptions about qualitative information. For example, the upper age limit for “children” can vary from 15 years to 21 years, “intense” physiotherapy might mean very different things to different researchers at different times and for different patients, and the volume of blood associated with “heavy” blood loss might vary widely depending on the setting.

#### Item 12: RISK OF BIAS IN INDIVIDUAL STUDIES

Describe methods used for assessing risk of bias in individual studies (including specification of whether this was done at the study or outcome level, or both), and how this information is to be used in any data synthesis.

**Example.** “To ascertain the validity of eligible randomized trials, pairs of reviewers working independently and with adequate reliability determined the adequacy of randomization and concealment of allocation, blinding of patients, health care providers, data collectors, and outcome assessors; and extent of loss to follow-up (i.e. proportion of patients in whom the investigators were not able to ascertain outcomes).” [85]

“To explore variability in study results (heterogeneity) we specified the following hypotheses before conducting the analysis. We hypothesised that effect size may differ according to the methodological quality of the studies.” [86]

#### Explanation

The likelihood that the treatment effect reported in a systematic review approximates the truth depends on the validity of the included studies, as certain methodological characteristics may be associated with effect sizes [87],[88]. For example, trials without reported adequate allocation concealment exaggerate treatment effects on average compared to those with adequate concealment [88]. Therefore, it is important for authors to describe any methods that they used to gauge the risk of bias in the included studies and how that information was used [89]. Additionally, authors should provide a rationale if no assessment of risk of bias was undertaken. The most popular term to describe the issues relevant to this item is “quality,” but for the reasons that are elaborated in Box 4 we prefer to name this item as “assessment of risk of bias.”

**Box 4. Study Quality and Risk of Bias**

In this paper, and elsewhere [11], we sought to use a new term for many readers, namely, risk of bias, for evaluating each included study in a systematic review. Previous papers [89],[188] tended to use the term “quality”. When carrying out a systematic review we believe it is important to distinguish between quality and risk of bias and to focus on evaluating and reporting the latter. Quality is often the best the authors have been able to do. For example, authors may report the results of surgical trials in which blinding of the outcome assessors was not part of the trial's conduct. Even though this may have been the best methodology the researchers were able to do, there are still theoretical grounds for believing that the study was susceptible to (risk of) bias.

Assessing the risk of bias should be part of the conduct and reporting of any systematic review. In all situations, we encourage systematic reviewers to think ahead carefully about what risks of bias (methodological and clinical) may have a bearing on the results of their systematic reviews.

For systematic reviewers, understanding the risk of bias on the results of studies is often difficult, because the report is only a surrogate of the actual conduct of the study. There is some suggestion [189],[190] that the report may not be a reasonable facsimile of the study, although this view is not shared by all [88],[191]. There are three main ways to assess risk of bias: individual components, checklists, and scales. There are a great many scales available [192], although we caution their use based on theoretical grounds [193] and emerging empirical evidence [194]. Checklists are less frequently used and potentially run the same problems as scales. We advocate using a component approach and one that is based on domains for which there is good empirical evidence and perhaps strong clinical grounds. The new Cochrane risk of bias tool [11] is one such component approach.

The Cochrane risk of bias tool consists of five items for which there is empirical evidence for their biasing influence on the estimates of an intervention's effectiveness in randomized trials (sequence generation, allocation concealment, blinding, incomplete outcome data, and selective outcome reporting) and a catch-all item called “other sources of bias” [11]. There is also some consensus that these items can be applied for evaluation of studies across very diverse clinical areas [93]. Other risk of bias items may be topic or even study specific, i.e., they may stem from some peculiarity of the research topic or some special feature of the design of a specific study. These peculiarities need to be investigated on a case-by-case basis, based on clinical and methodological acumen, and there can be no general recipe. In all situations, systematic reviewers need to think ahead carefully about what aspects of study quality may have a bearing on the results.

Many methods exist to assess the overall risk of bias in included studies, including scales, checklists, and individual components [90],[91]. As discussed in Box 4, scales that numerically summarize multiple components into a single number are misleading and unhelpful [92],[93]. Rather, authors should specify the methodological components that they assessed. Common markers of validity for randomized trials include the following: appropriate generation of random allocation sequence [94]; concealment of the allocation sequence [93]; blinding of participants, health care providers, data collectors, and outcome adjudicators [95],[96],[97],[98]; proportion of patients lost to follow-up [99],[100]; stopping of trials early for benefit [101]; and whether the analysis followed the intention-to-treat principle [100],[102]. The ultimate decision regarding which methodological features to evaluate requires consideration of the strength of the empiric data, theoretical rationale, and the unique circumstances of the included studies.

Authors should report how they assessed risk of bias; whether it was in a blind manner; and if assessments were completed by more than one person, and if so, whether they were completed independently [103],[104]. Similarly, we encourage authors to report any calibration exercises among review team members that were done. Finally, authors need to report how their assessments of risk of bias are used subsequently in the data synthesis (see Item 16). Despite the often difficult task of assessing the risk of bias in included studies, authors are sometimes silent on what they did with the resultant assessments [89]. If authors exclude studies from the review or any subsequent analyses on the basis of the risk of bias, they should tell readers which studies they excluded and explain the reasons for those exclusions (see Item 6). Authors should also describe any planned sensitivity or subgroup analyses related to bias assessments (see Item 16).

#### Item 13: SUMMARY MEASURES

State the principal summary measures (e.g., risk ratio, difference in means).

**Examples.** “Relative risk of mortality reduction was the primary measure of treatment effect.” [105]

“The meta-analyses were performed by computing relative risks (RRs) using random-effects model. Quantitative analyses were performed on an intention-to-treat basis and were confined to data derived from the period of follow-up. RR and 95% confidence intervals for each side effect (and all side effects) were calculated.” [106]

“The primary outcome measure was the mean difference in log_10_ HIV-1 viral load comparing zinc supplementation to placebo…” [107]

#### Explanation

When planning a systematic review, it is generally desirable that authors pre-specify the outcomes of primary interest (see Item 5) as well as the intended summary effect measure for each outcome. The chosen summary effect measure may differ from that used in some of the included studies. If possible the choice of effect measures should be explained, though it is not always easy to judge in advance which measure is the most appropriate.

For binary outcomes, the most common summary measures are the risk ratio, odds ratio, and risk difference [108]. Relative effects are more consistent across studies than absolute effects [109],[110], although absolute differences are important when interpreting findings (see Item 24).

For continuous outcomes, the natural effect measure is the difference in means [108]. Its use is appropriate when outcome measurements in all studies are made on the same scale. The standardized difference in means is used when the studies do not yield directly comparable data. Usually this occurs when all studies assess the same outcome but measure it in a variety of ways (e.g., different scales to measure depression).

For time-to-event outcomes, the hazard ratio is the most common summary measure. Reviewers need the log hazard ratio and its standard error for a study to be included in a meta-analysis [111]. This information may not be given for all studies, but methods are available for estimating the desired quantities from other reported information [111]. Risk ratio and odds ratio (in relation to events occurring by a fixed time) are not equivalent to the hazard ratio, and median survival times are not a reliable basis for meta-analysis [112]. If authors have used these measures they should describe their methods in the report.

#### Item 14: PLANNED METHODS OF ANALYSIS

Describe the methods of handling data and combining results of studies, if done, including measures of consistency (e.g., I^2^) for each meta-analysis.

**Examples.** “We tested for heterogeneity with the Breslow-Day test, and used the method proposed by Higgins et al. to measure inconsistency (the percentage of total variation across studies due to heterogeneity) of effects across lipid-lowering interventions. The advantages of this measure of inconsistency (termed I^2^) are that it does not inherently depend on the number of studies and is accompanied by an uncertainty interval.” [113]

“In very few instances, estimates of baseline mean or mean QOL [Quality of life] responses were obtained without corresponding estimates of variance (standard deviation [SD] or standard error). In these instances, an SD was imputed from the mean of the known SDs. In a number of cases, the response data available were the mean and variance in a pre study condition and after therapy. The within-patient variance in these cases could not be calculated directly and was approximated by assuming independence.” [114]

#### Explanation

The data extracted from the studies in the review may need some transformation (processing) before they are suitable for analysis or for presentation in an evidence table. Although such data handling may facilitate meta-analyses, it is sometimes needed even when meta-analyses are not done. For example, in trials with more than two intervention groups it may be necessary to combine results for two or more groups (e.g., receiving similar but non-identical interventions), or it may be desirable to include only a subset of the data to match the review's inclusion criteria. When several different scales (e.g., for depression) are used across studies, the sign of some scores may need to be reversed to ensure that all scales are aligned (e.g., so low values represent good health on all scales). Standard deviations may have to be reconstructed from other statistics such as *p*-values and *t* statistics [115],[116], or occasionally they may be imputed from the standard deviations observed in other studies [117]. Time-to-event data also usually need careful conversions to a consistent format [111]. Authors should report details of any such data processing.

Statistical combination of data from two or more separate studies in a meta-analysis may be neither necessary nor desirable (see Box 5 and Item 21). Regardless of the decision to combine individual study results, authors should report how they planned to evaluate between-study variability (heterogeneity or inconsistency) (Box 6). The consistency of results across trials may influence the decision of whether to combine trial results in a meta-analysis.

**Box 5. Whether or Not To Combine Data**

Deciding whether or not to combine data involves statistical, clinical, and methodological considerations. The statistical decisions are perhaps the most technical and evidence-based. These are more thoroughly discussed in Box 6. The clinical and methodological decisions are generally based on discussions within the review team and may be more subjective.

Clinical considerations will be influenced by the question the review is attempting to address. Broad questions might provide more “license” to combine more disparate studies, such as whether “Ritalin is effective in increasing focused attention in people diagnosed with attention deficit hyperactivity disorder (ADHD).” Here authors might elect to combine reports of studies involving children and adults. If the clinical question is more focused, such as whether “Ritalin is effective in increasing classroom attention in previously undiagnosed ADHD children who have no comorbid conditions,” it is likely that different decisions regarding synthesis of studies are taken by authors. In any case authors should describe their clinical decisions in the systematic review report.

Deciding whether or not to combine data also has a methodological component. Reviewers may decide not to combine studies of low risk of bias with those of high risk of bias (see Items 12 and 19). For example, for subjective outcomes, systematic review authors may not wish to combine assessments that were completed under blind conditions with those that were not.

For any particular question there may not be a “right” or “wrong” choice concerning synthesis, as such decisions are likely complex. However, as the choice may be subjective, authors should be transparent as to their key decisions and describe them for readers.

**Box 6. Meta-Analysis and Assessment of Consistency (Heterogeneity)**

**Meta-Analysis: Statistical Combination of the Results of Multiple Studies**

If it is felt that studies should have their results combined statistically, other issues must be considered because there are many ways to conduct a meta-analysis. Different effect measures can be used for both binary and continuous outcomes (see Item 13). Also, there are two commonly used statistical models for combining data in a meta-analysis [195]. The fixed-effect model assumes that there is a common treatment effect for all included studies [196]; it is assumed that the observed differences in results across studies reflect random variation [196]. The random-effects model assumes that there is no common treatment effect for all included studies but rather that the variation of the effects across studies follows a particular distribution [197]. In a random-effects model it is believed that the included studies represent a random sample from a larger population of studies addressing the question of interest [198].

There is no consensus about whether to use fixed- or random-effects models, and both are in wide use. The following differences have influenced some researchers regarding their choice between them. The random-effects model gives more weight to the results of smaller trials than does the fixed-effect analysis, which may be undesirable as small trials may be inferior and most prone to publication bias. The fixed-effect model considers only within-study variability whereas the random-effects model considers both within- and between-study variability. This is why a fixed-effect analysis tends to give narrower confidence intervals (i.e., provide greater precision) than a random-effects analysis [110],[196],[199]. In the absence of any between-study heterogeneity, the fixed- and random-effects estimates will coincide.

In addition, there are different methods for performing both types of meta-analysis [200]. Common fixed-effect approaches are Mantel-Haenszel and inverse variance, whereas random-effects analyses usually use the DerSimonian and Laird approach, although other methods exist, including Bayesian meta-analysis [201].

In the presence of demonstrable between-study heterogeneity (see below), some consider that the use of a fixed-effect analysis is counterintuitive because their main assumption is violated. Others argue that it is inappropriate to conduct any meta-analysis when there is unexplained variability across trial results. If the reviewers decide not to combine the data quantitatively, a danger is that eventually they may end up using quasi-quantitative rules of poor validity (e.g., vote counting of how many studies have nominally significant results) for interpreting the evidence. Statistical methods to combine data exist for almost any complex situation that may arise in a systematic review, but one has to be aware of their assumptions and limitations to avoid misapplying or misinterpreting these methods.

**Assessment of Consistency (Heterogeneity)**

We expect some variation (inconsistency) in the results of different studies due to chance alone. Variability in excess of that due to chance reflects true differences in the results of the trials, and is called “heterogeneity.” The conventional statistical approach to evaluating heterogeneity is a chi-squared test (Cochran's Q), but it has low power when there are few studies and excessive power when there are many studies [202]. By contrast, the I^2^ statistic quantifies the amount of variation in results across studies beyond that expected by chance and so is preferable to Q [202],[203]. I^2^ represents the percentage of the total variation in estimated effects across studies that is due to heterogeneity rather than to chance; some authors consider an I^2^ value less than 25% as low [202]. However, I^2^ also suffers from large uncertainty in the common situation where only a few studies are available [204], and reporting the uncertainty in I^2^ (e.g., as the 95% confidence interval) may be helpful [145]. When there are few studies, inferences about heterogeneity should be cautious.

When considerable heterogeneity is observed, it is advisable to consider possible reasons [205]. In particular, the heterogeneity may be due to differences between subgroups of studies (see Item 16). Also, data extraction errors are a common cause of substantial heterogeneity in results with continuous outcomes [139].

When meta-analysis is done, authors should specify the effect measure (e.g., relative risk or mean difference) (see Item 13), the statistical method (e.g., inverse variance), and whether a fixed- or random-effects approach, or some other method (e.g., Bayesian) was used (see Box 6). If possible, authors should explain the reasons for those choices.

#### Item 15: RISK OF BIAS ACROSS STUDIES

Specify any assessment of risk of bias that may affect the cumulative evidence (e.g., publication bias, selective reporting within studies).

**Examples.** “For each trial we plotted the effect by the inverse of its standard error. The symmetry of such ‘funnel plots’ was assessed both visually, and formally with Egger's test, to see if the effect decreased with increasing sample size.” [118]

“We assessed the possibility of publication bias by evaluating a funnel plot of the trial mean differences for asymmetry, which can result from the non publication of small trials with negative results…Because graphical evaluation can be subjective, we also conducted an adjusted rank correlation test and a regression asymmetry test as formal statistical tests for publication bias…We acknowledge that other factors, such as differences in trial quality or true study heterogeneity, could produce asymmetry in funnel plots.” [119]

#### Explanation

Reviewers should explore the possibility that the available data are biased. They may examine results from the available studies for clues that suggest there may be missing studies (publication bias) or missing data from the included studies (selective reporting bias) (see Box 7). Authors should report in detail any methods used to investigate possible bias across studies.

**Box 7. Bias Caused by Selective Publication of Studies or Results within Studies**

Systematic reviews aim to incorporate information from all relevant studies. The absence of information from some studies may pose a serious threat to the validity of a review. Data may be incomplete because some studies were not published, or because of incomplete or inadequate reporting within a published article. These problems are often summarized as “publication bias” although in fact the bias arises from non-publication of full studies and selective publication of results in relation to their findings. Non-publication of research findings dependent on the actual results is an important risk of bias to a systematic review and meta-analysis.

**Missing Studies**

Several empirical investigations have shown that the findings from clinical trials are more likely to be published if the results are statistically significant (*p*<0.05) than if they are not [125],[206],[207]. For example, of 500 oncology trials with more than 200 participants for which preliminary results were presented at a conference of the American Society of Clinical Oncology, 81% with *p*<0.05 were published in full within five years compared to only 68% of those with *p*>0.05 [208].

Also, among published studies, those with statistically significant results are published sooner than those with non-significant findings [209]. When some studies are missing for these reasons, the available results will be biased towards exaggerating the effect of an intervention.

**Missing Outcomes**

In many systematic reviews only some of the eligible studies (often a minority) can be included in a meta-analysis for a specific outcome. For some studies, the outcome may not be measured or may be measured but not reported. The former will not lead to bias, but the latter could.

Evidence is accumulating that selective reporting bias is widespread and of considerable importance [42],[43]. In addition, data for a given outcome may be analyzed in multiple ways and the choice of presentation influenced by the results obtained. In a study of 102 randomized trials, comparison of published reports with trial protocols showed that a median of 38% efficacy and 50% safety outcomes per trial, respectively, were not available for meta-analysis. Statistically significant outcomes had a higher odds of being fully reported in publications when compared with non-significant outcomes for both efficacy (pooled odds ratio 2.4; 95% confidence interval 1.4 to 4.0) and safety (4.7, 1.8 to 12) data. Several other studies have had similar findings [210],[211].

**Detection of Missing Information**

Missing studies may increasingly be identified from trials registries. Evidence of missing outcomes may come from comparison with the study protocol, if available, or by careful examination of published articles [11]. Study publication bias and selective outcome reporting are difficult to exclude or verify from the available results, especially when few studies are available.

If the available data are affected by either (or both) of the above biases, smaller studies would tend to show larger estimates of the effects of the intervention. Thus one possibility is to investigate the relation between effect size and sample size (or more specifically, precision of the effect estimate). Graphical methods, especially the funnel plot [212], and analytic methods (e.g., Egger's test) are often used [213],[214],[215], although their interpretation can be problematic [216],[217]. Strictly speaking, such analyses investigate “small study bias”; there may be many reasons why smaller studies have systematically different effect sizes than larger studies, of which reporting bias is just one [218]. Several alternative tests for bias have also been proposed, beyond the ones testing small study bias [215],[219],[220], but none can be considered a gold standard. Although evidence that smaller studies had larger estimated effects than large ones may suggest the possibility that the available evidence is biased, misinterpretation of such data is common [123].

It is difficult to assess whether within-study selective reporting is present in a systematic review. If a protocol of an individual study is available, the outcomes in the protocol and the published report can be compared. Even in the absence of a protocol, outcomes listed in the methods section of the published report can be compared with those for which results are presented [120]. In only half of 196 trial reports describing comparisons of two drugs in arthritis were all the effect variables in the methods and results sections the same [82]. In other cases, knowledge of the clinical area may suggest that it is likely that the outcome was measured even if it was not reported. For example, in a particular disease, if one of two linked outcomes is reported but the other is not, then one should question whether the latter has been selectively omitted [121],[122].

Only 36% (76 of 212) of therapeutic systematic reviews published in November 2004 reported that study publication bias was considered, and only a quarter of those intended to carry out a formal assessment for that bias [3]. Of 60 meta-analyses in 24 articles published in 2005 in which formal assessments were reported, most were based on fewer than ten studies; most displayed statistically significant heterogeneity; and many reviewers misinterpreted the results of the tests employed [123]. A review of trials of antidepressants found that meta-analysis of only the published trials gave effect estimates 32% larger on average than when all trials sent to the drug agency were analyzed [67].

#### Item 16: ADDITIONAL ANALYSES

Describe methods of additional analyses (e.g., sensitivity or subgroup analyses, meta-regression), if done, indicating which were pre-specified.

**Example.** “Sensitivity analyses were pre-specified. The treatment effects were examined according to quality components (concealed treatment allocation, blinding of patients and caregivers, blinded outcome assessment), time to initiation of statins, and the type of statin. One post-hoc sensitivity analysis was conducted including unpublished data from a trial using cerivastatin.” [124]

#### Explanation

Authors may perform additional analyses to help understand whether the results of their review are robust, all of which should be reported. Such analyses include sensitivity analysis, subgroup analysis, and meta-regression [125].

Sensitivity analyses are used to explore the degree to which the main findings of a systematic review are affected by changes in its methods or in the data used from individual studies (e.g., study inclusion criteria, results of risk of bias assessment). Subgroup analyses address whether the summary effects vary in relation to specific (usually clinical) characteristics of the included studies or their participants. Meta-regression extends the idea of subgroup analysis to the examination of the quantitative influence of study characteristics on the effect size [126]. Meta-regression also allows authors to examine the contribution of different variables to the heterogeneity in study findings. Readers of systematic reviews should be aware that meta-regression has many limitations, including a danger of over-interpretation of findings [127],[128].

Even with limited data, many additional analyses can be undertaken. The choice of which analysis to undertake will depend on the aims of the review. None of these analyses, however, are exempt from producing potentially misleading results. It is important to inform readers whether these analyses were performed, their rationale, and which were pre-specified.

### RESULTS

#### Item 17: STUDY SELECTION

Give numbers of studies screened, assessed for eligibility, and included in the review, with reasons for exclusions at each stage, ideally with a flow diagram.

**Examples.** *In text*:

“A total of 10 studies involving 13 trials were identified for inclusion in the review. The search of Medline, PsycInfo and Cinahl databases provided a total of 584 citations. After adjusting for duplicates 509 remained. Of these, 479 studies were discarded because after reviewing the abstracts it appeared that these papers clearly did not meet the criteria. Three additional studies…were discarded because full text of the study was not available or the paper could not be feasibly translated into English. The full text of the remaining 27 citations was examined in more detail. It appeared that 22 studies did not meet the inclusion criteria as described. Five studies…met the inclusion criteria and were included in the systematic review. An additional five studies…that met the criteria for inclusion were identified by checking the references of located, relevant papers and searching for studies that have cited these papers. No unpublished relevant studies were obtained.” [129]

**Figure 2:**
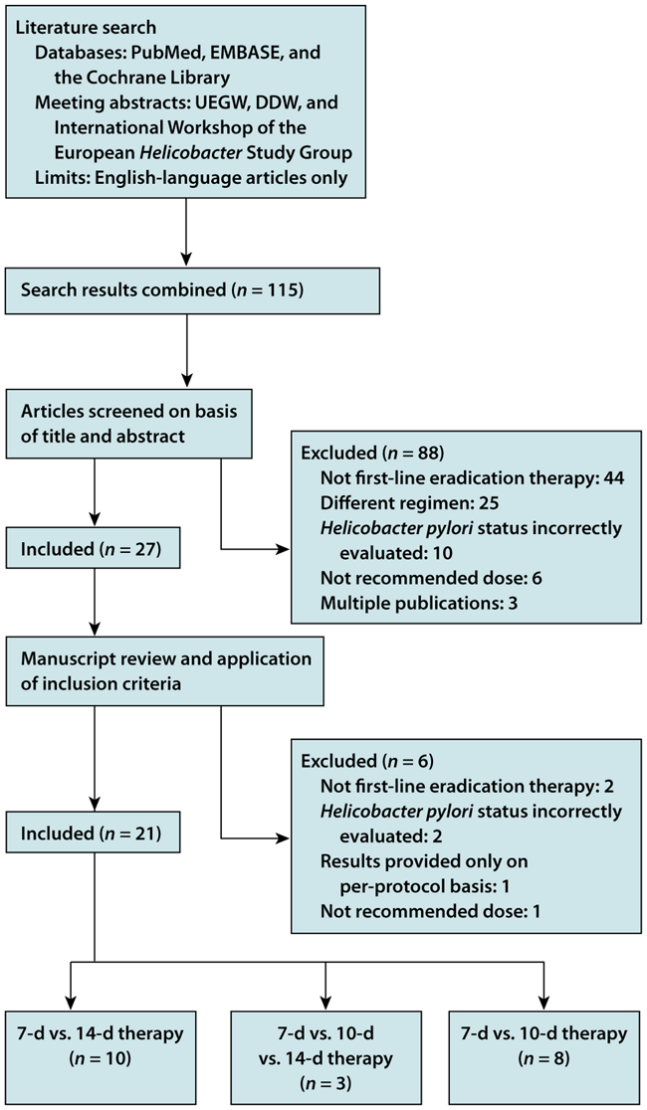
Example Figure: Example flow diagram of study selection. DDW, Digestive Disease Week; UEGW, United European Gastroenterology Week. Reproduced with permission from [130].

See flow diagram Figure 2.

#### Explanation

Authors should report, ideally with a flow diagram, the total number of records identified from electronic bibliographic sources (including specialized database or registry searches), hand searches of various sources, reference lists, citation indices, and experts. It is useful if authors delineate for readers the number of selected articles that were identified from the different sources so that they can see, for example, whether most articles were identified through electronic bibliographic sources or from references or experts. Literature identified primarily from references or experts may be prone to citation or publication bias [131],[132].

The flow diagram and text should describe clearly the process of report selection throughout the review. Authors should report: unique records identified in searches; records excluded after preliminary screening (e.g., screening of titles and abstracts); reports retrieved for detailed evaluation; potentially eligible reports that were not retrievable; retrieved reports that did not meet inclusion criteria and the primary reasons for exclusion; and the studies included in the review. Indeed, the most appropriate layout may vary for different reviews.

Authors should also note the presence of duplicate or supplementary reports so that readers understand the number of individual studies compared to the number of reports that were included in the review. Authors should be consistent in their use of terms, such as whether they are reporting on counts of citations, records, publications, or studies. We believe that reporting the number of studies is the most important.

A flow diagram can be very useful; it should depict all the studies included based upon fulfilling the eligibility criteria, whether or not data have been combined for statistical analysis. A recent review of 87 systematic reviews found that about half included a QUOROM flow diagram [133]. The authors of this research recommended some important ways that reviewers can improve the use of a flow diagram when describing the flow of information throughout the review process, including a separate flow diagram for each important outcome reported [133].

#### Item 18: STUDY CHARACTERISTICS

For each study, present characteristics for which data were extracted (e.g., study size, PICOS, follow-up period) and provide the citation.

**Examples.** *In text*:

“Characteristics of included studies

*Methods*

All four studies finally selected for the review were randomised controlled trials published in English. The duration of the intervention was 24 months for the RIO-North America and 12 months for the RIO-Diabetes, RIO-Lipids and RIO-Europe study. Although the last two described a period of 24 months during which they were conducted, only the first 12-months results are provided. All trials had a run-in, as a single blind period before the randomisation.

*Participants*

The included studies involved 6625 participants. The main inclusion criteria entailed adults (18 years or older), with a body mass index greater than 27 kg/m^2^ and less than 5 kg variation in body weight within the three months before study entry.

*Intervention*

All trials were multicentric. The RIO-North America was conducted in the USA and Canada, RIO-Europe in Europe and the USA, RIO-Diabetes in the USA and 10 other different countries not specified, and RIO-Lipids in eight unspecified different countries.

The intervention received was placebo, 5 mg of rimonabant or 20 mg of rimonabant once daily in addition to a mild hypocaloric diet (600 kcal/day deficit).

*Outcomes*

*Primary*

In all studies the primary outcome assessed was weight change from baseline after one year of treatment and the RIO-North America study also evaluated the prevention of weight regain between the first and second year. All studies evaluated adverse effects, including those of any kind and serious events. Quality of life was measured in only one study, but the results were not described (RIO-Europe).

*Secondary and additional outcomes*

These included prevalence of metabolic syndrome after one year and change in cardiometabolic risk factors such as blood pressure, lipid profile, etc.

No study included mortality and costs as outcome.

The timing of outcome measures was variable and could include monthly investigations, evaluations every three months or a single final evaluation after one year.” [134]

*In table*: See Table 2.

**Table 2:** Example Table: Summary of included studies evaluating the efficacy of antiemetic agents in acute gastroenteritis.

Source	Setting	No. of Patients	Age Range	Inclusion Criteria	Antiemetic Agent	Route	Follow-Up
Freedman et al., 2006	ED	214	6 months–10 years	GE with mild to moderate dehydration and vomiting in the preceding 4 hours	Ondansetron	PO	1–2 weeks
Reeves et al., 2002	ED	107	1 month–22 years	GE and vomiting requiring IV rehydration	Ondansetron	IV	5–7 days
Roslund et al., 2007	ED	106	1–10 years	GE with failed oral rehydration attempt in ED	Ondansetron	PO	1 week
Stork et al., 2006	ED	137	6 months–12 years	GE, recurrent emesis, mild to moderate dehydration, and failed oral hydration	Ondansetron and dexamethasone	IV	1 and 2 days

ED, emergency department; GE, gastroenteritis; IV, intravenous; PO, by mouth.

Adapted from [135].

#### Explanation

For readers to gauge the validity and applicability of a systematic review's results, they need to know something about the included studies. Such information includes PICOS (Box 2) and specific information relevant to the review question. For example, if the review is examining the long-term effects of antidepressants for moderate depressive disorder, authors should report the follow-up periods of the included studies. For each included study, authors should provide a citation for the source of their information regardless of whether or not the study is published. This information makes it easier for interested readers to retrieve the relevant publications or documents.

Reporting study-level data also allows the comparison of the main characteristics of the studies included in the review. Authors should present enough detail to allow readers to make their own judgments about the relevance of included studies. Such information also makes it possible for readers to conduct their own subgroup analyses and interpret subgroups, based on study characteristics.

Authors should avoid, whenever possible, assuming information when it is missing from a study report (e.g., sample size, method of randomization). Reviewers may contact the original investigators to try to obtain missing information or confirm the data extracted for the systematic review. If this information is not obtained, this should be noted in the report. If information is imputed, the reader should be told how this was done and for which items. Presenting study-level data makes it possible to clearly identify unpublished information obtained from the original researchers and make it available for the public record.

Typically, study-level characteristics are presented as a table as in the example in Table 2. Such presentation ensures that all pertinent items are addressed and that missing or unclear information is clearly indicated. Although paper-based journals do not generally allow for the quantity of information available in electronic journals or Cochrane reviews, this should not be accepted as an excuse for omission of important aspects of the methods or results of included studies, since these can, if necessary, be shown on a Web site.

Following the presentation and description of each included study, as discussed above, reviewers usually provide a narrative summary of the studies. Such a summary provides readers with an overview of the included studies. It may for example address the languages of the published papers, years of publication, and geographic origins of the included studies.

The PICOS framework is often helpful in reporting the narrative summary indicating, for example, the clinical characteristics and disease severity of the participants and the main features of the intervention and of the comparison group. For non-pharmacological interventions, it may be helpful to specify for each study the key elements of the intervention received by each group. Full details of the interventions in included studies were reported in only three of 25 systematic reviews relevant to general practice [84].

#### Item 19: RISK OF BIAS WITHIN STUDIES

Present data on risk of bias of each study and, if available, any outcome-level assessment (see Item 12).

**Example.** See Table 3.

**Table 3:** Example Table: Quality measures of the randomized controlled trials that failed to fulfill any one of six markers of validity.

Trials	Concealment of Randomisation	RCT Stopped Early	Patients Blinded	Health Care Providers Blinded	Data Collectors Blinded	Outcome Assessors Blinded
Liu	No	No	Yes	Yes	Yes	Yes
Stone	Yes	No	No	Yes	Yes	Yes
Polderman	Yes	Yes	No	No	No	Yes
Zaugg	Yes	No	No	No	Yes	Yes
Urban	Yes	Yes	No	No, except anesthesiologists	Yes	Yes

RCT, randomized controlled trial.

Adapted from [96].

#### Explanation

We recommend that reviewers assess the risk of bias in the included studies using a standard approach with defined criteria (see Item 12). They should report the results of any such assessments [89].

Reporting only summary data (e.g., “two of eight trials adequately concealed allocation”) is inadequate because it fails to inform readers which studies had the particular methodological shortcoming. A more informative approach is to explicitly report the methodological features evaluated for each study. The Cochrane Collaboration's new tool for assessing the risk of bias also requests that authors substantiate these assessments with any relevant text from the original studies [11]. It is often easiest to provide these data in a tabular format, as in the example. However, a narrative summary describing the tabular data can also be helpful for readers.

#### Item 20: RESULTS OF INDIVIDUAL STUDIES

For all outcomes considered (benefits and harms), present, for each study: (a) simple summary data for each intervention group and (b) effect estimates and confidence intervals, ideally with a forest plot.

**Figure 3:**
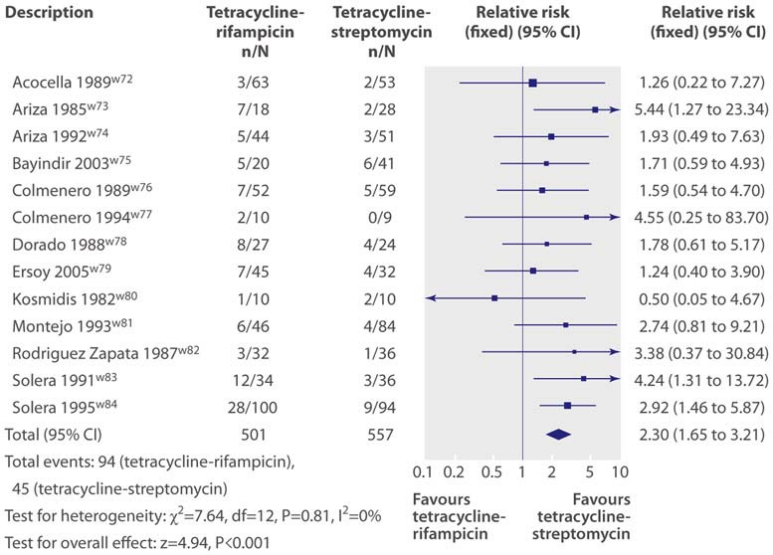
Example Figure: Overall failure (defined as failure of assigned regimen or relapse) with tetracycline-rifampicin versus tetracycline-streptomycin. CI, confidence interval. Reproduced with permission from [137].

**Examples.** See Table 4 and Figure 3.

**Table 4:** Example Table: Heterotopic ossification in trials comparing radiotherapy to non-steroidal anti-inflammatory drugs after major hip procedures and fractures.

Author (Year)	Radiotherapy		NSAID	
Kienapfel (1999)	12/49	24.5%	20/55	36.4%
Sell (1998)	2/77	2.6%	18/77	23.4%
Kolbl (1997)	39/188	20.7%	18/113	15.9%
Kolbl (1998)	22/46	47.8%	6/54	11.1%
Moore (1998)	9/33	27.3%	18/39	46.2%
Bremen-Kuhne (1997)	9/19	47.4%	11/31	35.5%
Knelles (1997)	5/101	5.0%	46/183	25.4%

NSAID, non-steroidal anti-inflammatory drug.

Adapted from [136].

#### Explanation

Publication of summary data from individual studies allows the analyses to be reproduced and other analyses and graphical displays to be investigated. Others may wish to assess the impact of excluding particular studies or consider subgroup analyses not reported by the review authors. Displaying the results of each treatment group in included studies also enables inspection of individual study features. For example, if only odds ratios are provided, readers cannot assess the variation in event rates across the studies, making the odds ratio impossible to interpret [138]. Additionally, because data extraction errors in meta-analyses are common and can be large [139], the presentation of the results from individual studies makes it easier to identify errors. For continuous outcomes, readers may wish to examine the consistency of standard deviations across studies, for example, to be reassured that standard deviation and standard error have not been confused [138].

For each study, the summary data for each intervention group are generally given for binary outcomes as frequencies with and without the event (or as proportions such as 12/45). It is not sufficient to report event rates per intervention group as percentages. The required summary data for continuous outcomes are the mean, standard deviation, and sample size for each group. In reviews that examine time-to-event data, the authors should report the log hazard ratio and its standard error (or confidence interval) for each included study. Sometimes, essential data are missing from the reports of the included studies and cannot be calculated from other data but may need to be imputed by the reviewers. For example, the standard deviation may be imputed using the typical standard deviations in the other trials [116],[117] (see Item 14). Whenever relevant, authors should indicate which results were not reported directly and had to be estimated from other information (see Item 13). In addition, the inclusion of unpublished data should be noted.

For all included studies it is important to present the estimated effect with a confidence interval. This information may be incorporated in a table showing study characteristics or may be shown in a forest plot [140]. The key elements of the forest plot are the effect estimates and confidence intervals for each study shown graphically, but it is preferable also to include, for each study, the numerical group-specific summary data, the effect size and confidence interval, and the percentage weight (see second example [Figure 3]). For discussion of the results of meta-analysis, see Item 21.

In principle, all the above information should be provided for every outcome considered in the review, including both benefits and harms. When there are too many outcomes for full information to be included, results for the most important outcomes should be included in the main report with other information provided as a Web appendix. The choice of the information to present should be justified in light of what was originally stated in the protocol. Authors should explicitly mention if the planned main outcomes cannot be presented due to lack of information. There is some evidence that information on harms is only rarely reported in systematic reviews, even when it is available in the original studies [141]. Selective omission of harms results biases a systematic review and decreases its ability to contribute to informed decision making.

#### Item 21: SYNTHESES OF RESULTS

Present the main results of the review. If meta-analyses are done, include for each, confidence intervals and measures of consistency.

**Examples.** “Mortality data were available for all six trials, randomizing 311 patients and reporting data for 305 patients. There were no deaths reported in the three respiratory syncytial virus/severe bronchiolitis trials; thus our estimate is based on three trials randomizing 232 patients, 64 of whom died. In the pooled analysis, surfactant was associated with significantly lower mortality (relative risk = 0.7, 95% confidence interval = 0.4–0.97, P = 0.04). There was no evidence of heterogeneity (I^2^ = 0%)”. [142]

“Because the study designs, participants, interventions, and reported outcome measures varied markedly, we focused on describing the studies, their results, their applicability, and their limitations and on qualitative synthesis rather than meta-analysis.” [143]

“We detected significant heterogeneity within this comparison (I^2^ = 46.6%; χ^2^ = 13.11, df = 7; P = 0.07). Retrospective exploration of the heterogeneity identified one trial that seemed to differ from the others. It included only small ulcers (wound area less than 5 cm^2^). Exclusion of this trial removed the statistical heterogeneity and did not affect the finding of no evidence of a difference in healing rate between hydrocolloids and simple low adherent dressings (relative risk = 0.98, [95% confidence interval] 0.85 to 1.12; I^2^ = 0%).” [144]

#### Explanation

Results of systematic reviews should be presented in an orderly manner. Initial narrative descriptions of the evidence covered in the review (see Item 18) may tell readers important things about the study populations and the design and conduct of studies. These descriptions can facilitate the examination of patterns across studies. They may also provide important information about applicability of evidence, suggest the likely effects of any major biases, and allow consideration, in a systematic manner, of multiple explanations for possible differences of findings across studies.

If authors have conducted one or more meta-analyses, they should present the results as an estimated effect across studies with a confidence interval. It is often simplest to show each meta-analysis summary with the actual results of included studies in a forest plot (see Item 20) [140]. It should always be clear which of the included studies contributed to each meta-analysis. Authors should also provide, for each meta-analysis, a measure of the consistency of the results from the included studies such as I^2^ (heterogeneity; see Box 6); a confidence interval may also be given for this measure [145]. If no meta-analysis was performed, the qualitative inferences should be presented as systematically as possible with an explanation of why meta-analysis was not done, as in the second example above [143]. Readers may find a forest plot, without a summary estimate, helpful in such cases.

Authors should in general report syntheses for all the outcome measures they set out to investigate (i.e., those described in the protocol; see Item 4) to allow readers to draw their own conclusions about the implications of the results. Readers should be made aware of any deviations from the planned analysis. Authors should tell readers if the planned meta-analysis was not thought appropriate or possible for some of the outcomes and the reasons for that decision.

It may not always be sensible to give meta-analysis results and forest plots for each outcome. If the review addresses a broad question, there may be a very large number of outcomes. Also, some outcomes may have been reported in only one or two studies, in which case forest plots are of little value and may be seriously biased.

Of 300 systematic reviews indexed in MEDLINE in 2004, a little more than half (54%) included meta-analyses, of which the majority (91%) reported assessing for inconsistency in results.

#### Item 22: RISK OF BIAS ACROSS STUDIES

Present results of any assessment of risk of bias across studies (see Item 15).

**Figure 4:**
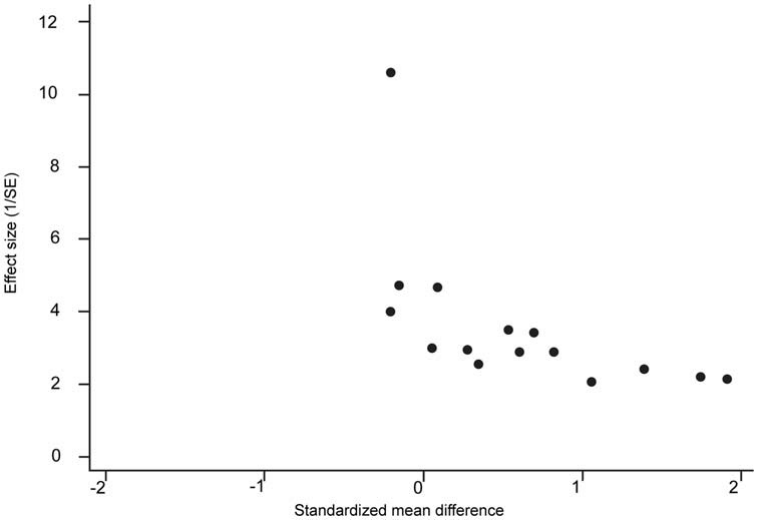
Example Figure: Example of a funnel plot showing evidence of considerable asymmetry. SE, standard error. Adapted from [146], with permission.

**Examples.** “Strong evidence of heterogeneity (I^2^ = 79%, *P*<0.001) was observed. To explore this heterogeneity, a funnel plot was drawn. The funnel plot in Figure 4 shows evidence of considerable asymmetry.” [146]

“Specifically, four sertraline trials involving 486 participants and one citalopram trial involving 274 participants were reported as having failed to achieve a statistically significant drug effect, without reporting mean HRSD [Hamilton Rating Scale for Depression] scores. We were unable to find data from these trials on pharmaceutical company Web sites or through our search of the published literature. These omissions represent 38% of patients in sertraline trials and 23% of patients in citalopram trials. Analyses with and without inclusion of these trials found no differences in the patterns of results; similarly, the revealed patterns do not interact with drug type. The purpose of using the data obtained from the FDA was to avoid publication bias, by including unpublished as well as published trials. Inclusion of only those sertraline and citalopram trials for which means were reported to the FDA would constitute a form of reporting bias similar to publication bias and would lead to overestimation of drug–placebo differences for these drug types. Therefore, we present analyses only on data for medications for which complete clinical trials' change was reported.” [147]

#### Explanation

Authors should present the results of any assessments of risk of bias across studies. If a funnel plot is reported, authors should specify the effect estimate and measure of precision used, presented typically on the *x*-axis and *y*-axis, respectively. Authors should describe if and how they have tested the statistical significance of any possible asymmetry (see Item 15). Results of any investigations of selective reporting of outcomes within studies (as discussed in Item 15) should also be reported. Also, we advise authors to tell readers if any pre-specified analyses for assessing risk of bias across studies were not completed and the reasons (e.g., too few included studies).

#### Item 23: ADDITIONAL ANALYSES

Give results of additional analyses, if done (e.g., sensitivity or subgroup analyses, meta-regression [see Item 16]).

**Examples.** “…benefits of chondroitin were smaller in trials with adequate concealment of allocation compared with trials with unclear concealment (P for interaction = 0.050), in trials with an intention-to-treat analysis compared with those that had excluded patients from the analysis (P for interaction = 0.017), and in large compared with small trials (P for interaction = 0.022).” [148]

“Subgroup analyses according to antibody status, antiviral medications, organ transplanted, treatment duration, use of antilymphocyte therapy, time to outcome assessment, study quality and other aspects of study design did not demonstrate any differences in treatment effects. Multivariate meta-regression showed no significant difference in CMV [cytomegalovirus] disease after allowing for potential confounding or effect-modification by prophylactic drug used, organ transplanted or recipient serostatus in CMV positive recipients and CMV negative recipients of CMV positive donors.” [149]

#### Explanation

Authors should report any subgroup or sensitivity analyses and whether or not they were pre-specified (see Items 5 and 16). For analyses comparing subgroups of studies (e.g., separating studies of low- and high-dose aspirin), the authors should report any tests for interactions, as well as estimates and confidence intervals from meta-analyses within each subgroup. Similarly, meta-regression results (see Item 16) should not be limited to *p*-values, but should include effect sizes and confidence intervals [150], as the first example reported above does in a table. The amount of data included in each additional analysis should be specified if different from that considered in the main analyses. This information is especially relevant for sensitivity analyses that exclude some studies; for example, those with high risk of bias.

Importantly, all additional analyses conducted should be reported, not just those that were statistically significant. This information will help avoid selective outcome reporting bias within the review as has been demonstrated in reports of randomized controlled trials [42],[44],[121],[151],[152]. Results from exploratory subgroup or sensitivity analyses should be interpreted cautiously, bearing in mind the potential for multiple analyses to mislead.

### DISCUSSION

#### Item 24: SUMMARY OF EVIDENCE

Summarize the main findings, including the strength of evidence for each main outcome; consider their relevance to key groups (e.g., health care providers, users, and policy makers).

**Example.** “Overall, the evidence is not sufficiently robust to determine the comparative effectiveness of angioplasty (with or without stenting) and medical treatment alone. Only 2 randomized trials with long-term outcomes and a third randomized trial that allowed substantial crossover of treatment after 3 months directly compared angioplasty and medical treatment…the randomized trials did not evaluate enough patients or did not follow patients for a sufficient duration to allow definitive conclusions to be made about clinical outcomes, such as mortality and cardiovascular or kidney failure events.

Some acceptable evidence from comparison of medical treatment and angioplasty suggested no difference in long-term kidney function but possibly better blood pressure control after angioplasty, an effect that may be limited to patients with bilateral atherosclerotic renal artery stenosis. The evidence regarding other outcomes is weak. Because the reviewed studies did not explicitly address patients with rapid clinical deterioration who may need acute intervention, our conclusions do not apply to this important subset of patients.” [143]

#### Explanation

Authors should give a brief and balanced summary of the nature and findings of the review. Sometimes, outcomes for which little or no data were found should be noted due to potential relevance for policy decisions and future research. Applicability of the review's findings, to different patients, settings, or target audiences, for example, should be mentioned. Although there is no standard way to assess applicability simultaneously to different audiences, some systems do exist [153]. Sometimes, authors formally rate or assess the overall body of evidence addressed in the review and can present the strength of their summary recommendations tied to their assessments of the quality of evidence (e.g., the GRADE system) [10].

Authors need to keep in mind that statistical significance of the effects does not always suggest clinical or policy relevance. Likewise, a non-significant result does not demonstrate that a treatment is ineffective. Authors should ideally clarify trade-offs and how the values attached to the main outcomes would lead different people to make different decisions. In addition, adroit authors consider factors that are important in translating the evidence to different settings and that may modify the estimates of effects reported in the review [153]. Patients and health care providers may be primarily interested in which intervention is most likely to provide a benefit with acceptable harms, while policy makers and administrators may value data on organizational impact and resource utilization.

#### Item 25: LIMITATIONS

Discuss limitations at study and outcome level (e.g., risk of bias), and at review level (e.g., incomplete retrieval of identified research, reporting bias).

**Examples.** *Outcome level:*

“The meta-analysis reported here combines data across studies in order to estimate treatment effects with more precision than is possible in a single study. The main limitation of this meta-analysis, as with any overview, is that the patient population, the antibiotic regimen and the outcome definitions are not the same across studies.” [154]

*Study and review level:*

“Our study has several limitations. The quality of the studies varied. Randomization was adequate in all trials; however, 7 of the articles did not explicitly state that analysis of data adhered to the intention-to-treat principle, which could lead to overestimation of treatment effect in these trials, and we could not assess the quality of 4 of the 5 trials reported as abstracts. Analyses did not identify an association between components of quality and re-bleeding risk, and the effect size in favour of combination therapy remained statistically significant when we excluded trials that were reported as abstracts.

Publication bias might account for some of the effect we observed. Smaller trials are, in general, analyzed with less methodological rigor than larger studies, and an asymmetrical funnel plot suggests that selective reporting may have led to an overestimation of effect sizes in small trials.” [155]

#### Explanation

A discussion of limitations should address the validity (i.e., risk of bias) and reporting (informativeness) of the included studies, limitations of the review process, and generalizability (applicability) of the review. Readers may find it helpful if authors discuss whether studies were threatened by serious risks of bias, whether the estimates of the effect of the intervention are too imprecise, or if there were missing data for many participants or important outcomes.

Limitations of the review process might include limitations of the search (e.g., restricting to English-language publications), and any difficulties in the study selection, appraisal, and meta-analysis processes. For example, poor or incomplete reporting of study designs, patient populations, and interventions may hamper interpretation and synthesis of the included studies [84]. Applicability of the review may be affected if there are limited data for certain populations or subgroups where the intervention might perform differently or few studies assessing the most important outcomes of interest; or if there is a substantial amount of data relating to an outdated intervention or comparator or heavy reliance on imputation of missing values for summary estimates (Item 14).

#### Item 26: CONCLUSIONS

Provide a general interpretation of the results in the context of other evidence, and implications for future research.

**Example.** *Implications for practice:*

“Between 1995 and 1997 five different meta-analyses of the effect of antibiotic prophylaxis on infection and mortality were published. All confirmed a significant reduction in infections, though the magnitude of the effect varied from one review to another. The estimated impact on overall mortality was less evident and has generated considerable controversy on the cost effectiveness of the treatment. Only one among the five available reviews, however, suggested that a weak association between respiratory tract infections and mortality exists and lack of sufficient statistical power may have accounted for the limited effect on mortality.”

*Implications for research*:

“A logical next step for future trials would thus be the comparison of this protocol against a regimen of a systemic antibiotic agent only to see whether the topical component can be dropped. We have already identified six such trials but the total number of patients so far enrolled (n = 1056) is too small for us to be confident that the two treatments are really equally effective. If the hypothesis is therefore considered worth testing more and larger randomised controlled trials are warranted. Trials of this kind, however, would not resolve the relevant issue of treatment induced resistance. To produce a satisfactory answer to this, studies with a different design would be necessary. Though a detailed discussion goes beyond the scope of this paper, studies in which the intensive care unit rather than the individual patient is the unit of randomisation and in which the occurrence of antibiotic resistance is monitored over a long period of time should be undertaken.” [156]

#### Explanation

Systematic reviewers sometimes draw conclusions that are too optimistic [157] or do not consider the harms equally as carefully as the benefits, although some evidence suggests these problems are decreasing [158]. If conclusions cannot be drawn because there are too few reliable studies, or too much uncertainty, this should be stated. Such a finding can be as important as finding consistent effects from several large studies.

Authors should try to relate the results of the review to other evidence, as this helps readers to better interpret the results. For example, there may be other systematic reviews about the same general topic that have used different methods or have addressed related but slightly different questions [159],[160]. Similarly, there may be additional information relevant to decision makers, such as the cost-effectiveness of the intervention (e.g., health technology assessment). Authors may discuss the results of their review in the context of existing evidence regarding other interventions.

We advise authors also to make explicit recommendations for future research. In a sample of 2,535 Cochrane reviews, 82% included recommendations for research with specific interventions, 30% suggested the appropriate type of participants, and 52% suggested outcome measures for future research [161]. There is no corresponding assessment about systematic reviews published in medical journals, but we believe that such recommendations are much less common in those reviews.

Clinical research should not be planned without a thorough knowledge of similar, existing research [162]. There is evidence that this still does not occur as it should and that authors of primary studies do not consider a systematic review when they design their studies [163]. We believe systematic reviews have great potential for guiding future clinical research.

### FUNDING

#### Item 27: FUNDING

Describe sources of funding or other support (e.g., supply of data) for the systematic review; role of funders for the systematic review.

**Examples:** “The evidence synthesis upon which this article was based was funded by the Centers for Disease Control and Prevention for the Agency for Healthcare Research and Quality and the U.S. Prevention Services Task Force.” [164]

“Role of funding source: the funders played no role in study design, collection, analysis, interpretation of data, writing of the report, or in the decision to submit the paper for publication. They accept no responsibility for the contents.” [165]

#### Explanation

Authors of systematic reviews, like those of any other research study, should disclose any funding they received to carry out the review, or state if the review was not funded. Lexchin and colleagues [166] observed that outcomes of reports of randomized trials and meta-analyses of clinical trials funded by the pharmaceutical industry are more likely to favor the sponsor's product compared to studies with other sources of funding. Similar results have been reported elsewhere [167],[168]. Analogous data suggest that similar biases may affect the conclusions of systematic reviews [169].

Given the potential role of systematic reviews in decision making, we believe authors should be transparent about the funding and the role of funders, if any. Sometimes the funders will provide services, such as those of a librarian to complete the searches for relevant literature or access to commercial databases not available to the reviewers. Any level of funding or services provided to the systematic review team should be reported. Authors should also report whether the funder had any role in the conduct or report of the review. Beyond funding issues, authors should report any real or perceived conflicts of interest related to their role or the role of the funder in the reporting of the systematic review [170].

In a survey of 300 systematic reviews published in November 2004, funding sources were not reported in 41% of the reviews [3]. Only a minority of reviews (2%) reported being funded by for-profit sources, but the true proportion may be higher [171].

## Additional Considerations for Systematic Reviews of Non-Randomized Intervention Studies or for Other Types of Systematic Reviews

The PRISMA Statement and this document have focused on systematic reviews of reports of randomized trials. Other study designs, including non-randomized studies, quasi-experimental studies, and interrupted time series, are included in some systematic reviews that evaluate the effects of health care interventions [172],[173]. The methods of these reviews may differ to varying degrees from the typical intervention review, for example regarding the literature search, data abstraction, assessment of risk of bias, and analysis methods. As such, their reporting demands might also differ from what we have described here. A useful principle is for systematic review authors to ensure that their methods are reported with adequate clarity and transparency to enable readers to critically judge the available evidence and replicate or update the research.

In some systematic reviews, the authors will seek the raw data from the original researchers to calculate the summary statistics. These systematic reviews are called individual patient (or participant) data reviews [40],[41]. Individual patient data meta-analyses may also be conducted with prospective accumulation of data rather than retrospective accumulation of existing data. Here too, extra information about the methods will need to be reported.

Other types of systematic reviews exist. Realist reviews aim to determine how complex programs work in specific contexts and settings [174]. Meta-narrative reviews aim to explain complex bodies of evidence through mapping and comparing different over-arching storylines [175]. Network meta-analyses, also known as multiple treatments meta-analyses, can be used to analyze data from comparisons of many different treatments [176],[177]. They use both direct and indirect comparisons, and can be used to compare interventions that have not been directly compared.

We believe that the issues we have highlighted in this paper are relevant to ensure transparency and understanding of the processes adopted and the limitations of the information presented in systematic reviews of different types. We hope that PRISMA can be the basis for more detailed guidance on systematic reviews of other types of research, including diagnostic accuracy and epidemiological studies.

## Discussion

We developed the PRISMA Statement using an approach for developing reporting guidelines that has evolved over several years [178]. The overall aim of PRISMA is to help ensure the clarity and transparency of reporting of systematic reviews, and recent data indicate that this reporting guidance is much needed [3]. PRISMA is not intended to be a quality assessment tool and it should not be used as such.

This PRISMA Explanation and Elaboration document was developed to facilitate the understanding, uptake, and dissemination of the PRISMA Statement and hopefully provide a pedagogical framework for those interested in conducting and reporting systematic reviews. It follows a format similar to that used in other explanatory documents [17],[18],[19]. Following the recommendations in the PRISMA checklist may increase the word count of a systematic review report. We believe, however, that the benefit of readers being able to critically appraise a clear, complete, and transparent systematic review report outweighs the possible slight increase in the length of the report.

While the aims of PRISMA are to reduce the risk of flawed reporting of systematic reviews and improve the clarity and transparency in how reviews are conducted, we have little data to state more definitively whether this “intervention” will achieve its intended goal. A previous effort to evaluate QUOROM was not successfully completed [178]. Publication of the QUOROM Statement was delayed for two years while a research team attempted to evaluate its effectiveness by conducting a randomized controlled trial with the participation of eight major medical journals. Unfortunately that trial was not completed due to accrual problems (David Moher, personal communication). Other evaluation methods might be easier to conduct. At least one survey of 139 published systematic reviews in the critical care literature [179] suggests that their quality improved after the publication of QUOROM.

If the PRISMA Statement is endorsed by and adhered to in journals, as other reporting guidelines have been [17],[18],[19],[180], there should be evidence of improved reporting of systematic reviews. For example, there have been several evaluations of whether the use of CONSORT improves reports of randomized controlled trials. A systematic review of these studies [181] indicates that use of CONSORT is associated with improved reporting of certain items, such as allocation concealment. We aim to evaluate the benefits (i.e., improved reporting) and possible adverse effects (e.g., increased word length) of PRISMA and we encourage others to consider doing likewise.

Even though we did not carry out a systematic literature search to produce our checklist, and this is indeed a limitation of our effort, PRISMA was nevertheless developed using an evidence-based approach, whenever possible. Checklist items were included if there was evidence that not reporting the item was associated with increased risk of bias, or where it was clear that information was necessary to appraise the reliability of a review. To keep PRISMA up-to-date and as evidence-based as possible requires regular vigilance of the literature, which is growing rapidly. Currently the Cochrane Methodology Register has more than 11,000 records pertaining to the conduct and reporting of systematic reviews and other evaluations of health and social care. For some checklist items, such as reporting the abstract (Item 2), we have used evidence from elsewhere in the belief that the issue applies equally well to reporting of systematic reviews. Yet for other items, evidence does not exist; for example, whether a training exercise improves the accuracy and reliability of data extraction. We hope PRISMA will act as a catalyst to help generate further evidence that can be considered when further revising the checklist in the future.

More than ten years have passed between the development of the QUOROM Statement and its update, the PRISMA Statement. We aim to update PRISMA more frequently. We hope that the implementation of PRISMA will be better than it has been for QUOROM. There are at least two reasons to be optimistic. First, systematic reviews are increasingly used by health care providers to inform “best practice” patient care. Policy analysts and managers are using systematic reviews to inform health care decision making, and to better target future research. Second, we anticipate benefits from the development of the EQUATOR Network, described below.

Developing any reporting guideline requires considerable effort, experience, and expertise. While reporting guidelines have been successful for some individual efforts [17],[18],[19], there are likely others who want to develop reporting guidelines who possess little time, experience, or knowledge as to how to do so appropriately. The EQUATOR Network (Enhancing the QUAlity and Transparency Of health Research) aims to help such individuals and groups by serving as a global resource for anybody interested in developing reporting guidelines, regardless of the focus [7],[180],[182]. The overall goal of EQUATOR is to improve the quality of reporting of all health science research through the development and translation of reporting guidelines. Beyond this aim, the network plans to develop a large Web presence by developing and maintaining a resource center of reporting tools, and other information for reporting research (http://www.equator-network.org/).

We encourage health care journals and editorial groups, such as the World Association of Medical Editors and the International Committee of Medical Journal Editors, to endorse PRISMA in much the same way as they have endorsed other reporting guidelines, such as CONSORT. We also encourage editors of health care journals to support PRISMA by updating their “Instructions to Authors” and including the PRISMA Web address, and by raising awareness through specific editorial actions.

## Supporting Information

Figure S1 Flow of information through the different phases of a systematic review (downloadable template document for researchers to re-use).
(0.08 MB DOC)

Text S1 Checklist of items to include when reporting a systematic review or meta-analysis (downloadable template document for researchers to re-use).
(0.04 MB DOC)

## Abbreviations

PICOS: participants, interventions, comparators, outcomes, and study design
PRISMA: Preferred Reporting Items for Systematic reviews and Meta-Analyses
QUOROM: *QU*ality *O*f *R*eporting *O*f *M*eta-analyses
