# Supplementary material for: The PRISMA Statement for Reporting Systematic Reviews and Meta-Analyses of Studies That Evaluate Health Care Interventions: Explanation and Elaboration
Source: PLoS Med. 2009 Jul 21;6(7):e1000100. doi: 10.1371/journal.pmed.1000100 (PMC2707010; doi:10.1371/journal.pmed.1000100)
Supplement: Text S1 — Checklist of items to include when reporting a systematic review or meta-analysis (downloadable template document for researchers to re-use). (0.04 MB DOC) [file pmed.1000100.s002.doc]

**Identification**

**Screening**

**Eligibility**

**Included**

# of records identified through database searching

# of additional records identified through other sources

# of records after duplicates removed

# of records screened

# of records excluded

# of full-text articles assessed for eligibility

# of studies included in qualitative synthesis

# of full-text articles excluded, with reasons

# of studies included in quantitative synthesis (meta-analysis)
